# Phytochemical Profiling and Biological Activities of Extracts from Bioreactor-Grown Suspension Cell Cultures of *Schisandra henryi*

**DOI:** 10.3390/molecules29225260

**Published:** 2024-11-07

**Authors:** Karolina Jafernik, Adam Kokotkiewicz, Michał Dziurka, Aleksandra Kruk, Anna Hering, Krzysztof Jędrzejewski, Piotr Waligórski, Piotr Graczyk, Paweł Kubica, Justyna Stefanowicz-Hajduk, Sebastian Granica, Maria Łuczkiewicz, Agnieszka Szopa

**Affiliations:** 1 Department of Medicinal Plant and Mushroom Biotechnology, Jagiellonian University, Medical College, ul. Medyczna 9, 30-688 Kraków, Poland; karolina.jafernik@doctoral.uj.edu.pl (K.J.); p.kubica@uj.edu.pl (P.K.); 2 Department of Pharmacognosy, Medical University of Gdańsk, Al. Generała Józefa Hallera 107, 80-416 Gdańsk, Poland; adam.kokotkiewicz@gumed.edu.pl (A.K.); jedrzejewski.k.j@gumed.edu.pl (K.J.); maria.luczkiewicz@gumed.edu.pl (M.Ł.); 3 Polish Academy of Sciences, The Franciszek Górski Institute of Plant Physiology, ul. Niezapominajek 21, 30-239 Kraków, Poland; michal.dziurka@gmail.com (M.D.); p.waligorski@ifr-pan.edu.pl (P.W.); 4 Microbiota Lab, Department of Pharmaceutical Biology, Medical University of Warsaw, ul. Banacha 1, 02-097 Warszawa, Poland; aleksandra.kruk@wum.edu.pl (A.K.); sebastian.granica@wum.edu.pl (S.G.); 5 Department of Biology and Pharmaceutical Botany, Faculty of Pharmacy, Medical University of Gdańsk, Al. Generała Józefa Hallera 107, 80-416 Gdańsk, Poland; anna.hering@gumed.edu.pl (A.H.); piotr.graczyk@gumed.edu.pl (P.G.); justyna.stefanowicz-hajduk@gumed.edu.pl (J.S.-H.)

**Keywords:** *Schisandra henryi*, plant culture, phenolic compounds, dibenzocyclooctadiene lignans, antioxidant potential, cytotoxic activity, inhibition of hyaluronidase and tyrosinase

## Abstract

Plant biotechnology creates opportunities for the cultivation of plants regardless of their natural habitats, which are often protected or difficult to access. Maintaining suspension cell cultures in bioreactors is an advanced part of biotechnological research that provides possibilities for obtaining plant tissue on a large scale. In this study, the suspension culture cultivation of a Chinese endemic plant, *Schisandra henryi*, in a stirred tank bioreactor was elaborated for the first time. The phytochemical profile of the tissue extracts was determined with UHPLC-MS/MS for the lignans (fifteen dibenzocyclooctadiene lignans, one aryltetralin lignan, and two neolignans) and UHPLC-DAD-ESI-MS^3^ for the phenolic compounds (procyanidins and their derivatives and catechin). The maximum total lignan content of 1289 µg/100 g DW was detected for the extracts from suspensions cultured in a bioreactor for over 10 days. For the phenolic compounds, catechin was the dominant compound (390.44 mg/100 g DW). The biological activity of the extracts was tested too. To determine antioxidant potential we used DPPH (2,2-diphenyl-1-picrylhydrazyl), ABTS (2,2′-azino-bis(3-ethylbenzothiazoline-6-sulfonic acid), Molybdenum reduction, and β-carotene bleaching tests. The inhibition activity of the *S. henryi* extract on the enzymes responsible for skin aging, hyaluronidase and tyrosinase, was assessed with spectrophotometry. The cytotoxic activity of the extracts was estimated on human ovarian SKOV-3, cervical HeLa, and gastric AGS cancer cells and non-cancer, normal fibroblasts by an MTT (3-(4,5-dimethylthiazol-2-yl)-2,5-diphenyltetrazolium bromide) assay. The results showed the great potential of the obtained cell biomass extracts. The results of the antioxidant tests indicated their strong ability to reduce the level of free radicals, similarly to that of ascorbic acid, as well as the weak capacity to protect lipids from oxidation. Moreover, anticancer potential, particularly on the cervical and gastric cancer cells, was confirmed too.

## 1. Introduction

Plant biotechnology is one of the fastest developing fields of science. Some individual aspects include focusing on improving tissue multiplication processes and increasing the production of plant secondary metabolites [1,2,3,4]. Another one of them is conducting in vitro cultures in various types of bioreactor in order to accelerate tissue multiplication [4,5].

Suspension cell cultures are characterized by many advantages over microshoot cultures. Conducting a cell suspension culture in bioreactors is becoming an increasingly popular method of obtaining secondary metabolites. Large-scale production provides a number of possibilities and facilitates the acquisition of larger quantities of selected active substances. The growth of cell suspension biomass is relatively faster compared to that of microshoot cultures, adventitious roots, or hairy roots [6]. It has been shown that extracts from suspension cell cultures can be an alternative to obtaining, for example, valuable phenolic compounds like, e.g., verbascoside from *Verbena officinalis* [7], phytoestrogens from *Genista tinctoria* [8], or rosmarinic acid from *Lavandula officinalis* [6].

*Schisandra henryi* C.B. Clarke is an endemic species, occurring naturally only in Yunnan province in China, where there are specific conditions that enable the optimal growth of the plant (subtropical climate) [9,10]. *S. henryi* belongs to the Schisandraceae family and is a vine species that is not fully known in European countries. There is a small amount of research on *S. henryi* that focuses on the analysis of the chemical composition of stem extracts and cytotoxic activity [11]. Our team’s research on *S. henryi* focused on optimizing in vitro culture processes and analyzing the chemical composition of plant extracts [12,13]. Research show that the chemical profile of *S. henryi* is similar to that of another species from the Schisandraceae family, *Schisandra chinensis* (Turcz.) Baill., which is a pharmacopoeial species [14,15,16,17]. The raw material is fruit, which has, among others, anti-inflammatory, hepatoprotective, anticancer, and adaptogenic effects [18,19,20,21]. The main group of compounds responsible for the biological activities mentioned above is diebenzocyclooctadiene lignans. The presence of this group of metabolites, besides other groups of lignans, in *S. henryi* extracts has also been confirmed. Research on lignans shows that they have anti-inflammatory, antioxidant, hepatoprotective, and anticancer properties [21,22,23,24,25].

The aim of this research was to perform the initiation and optimization of *S. henryi* suspension cell culture cultivation in a stirred tank bioreactor and to analyze the chemical composition of the obtained extracts. Phytochemical tests were carried out using the UHPLC-MS/MS method for the qualitative and quantitative determinations of lignans and using UHPLC-DAD-ESI-MS^3^ and HPLC-DAD for qualitative and quantitative determinations of the phenolic compounds. Additionally, biological activity was tested for the first time, including antioxidant activity using DPPH, ABTS, Molybdenum reduction, and β-carotene bleaching tests, inhibiting the activity of enzymes, hyaluronidase and tyrosinase, as well as cytotoxic activity on human ovarian SKOV-3, cervical HeLa, and gastric AGS cancer cells and non-cancer, normal fibroblasts.

## 2. Results

### 2.1. The Suspension Culture Grown in Stirred Tank Bioreactor

**Figure 1:**
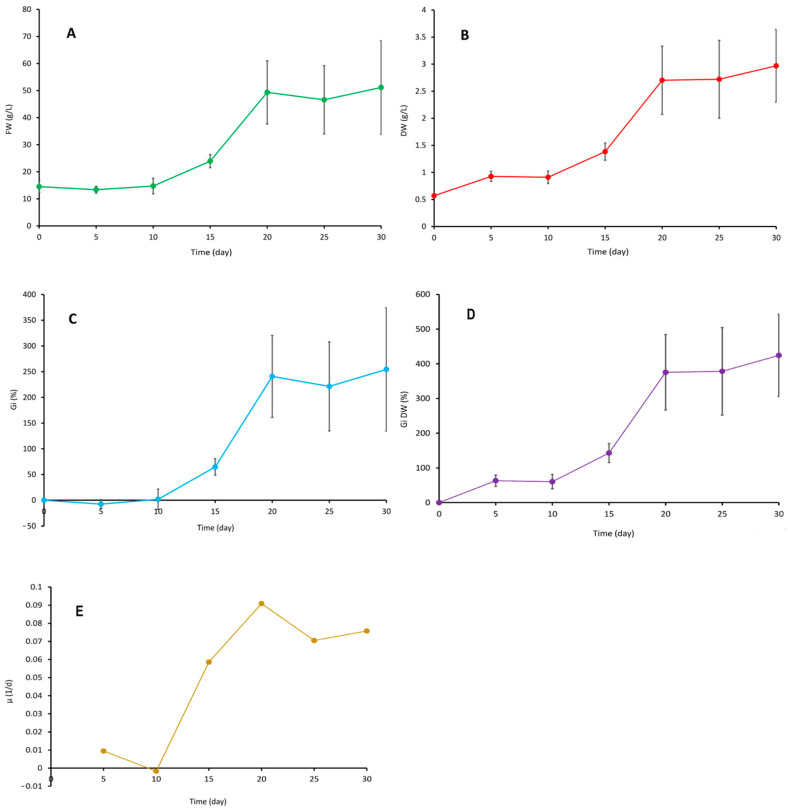
The fresh weight (**A**), dry weight (**B**), growth index (**C**), DW growth index (**D**) and specific growth rate (**E**) profiles of the *Schisandra henryi* cell suspension cultures grown in the stirred tank bioreactor for 30 days. The values are the means of 4 repetitions +/− SD. The growth indices were calculate using this formula, Gi = [(FWx − FW0)/FW0] × 100%, where FWx is the amount of fresh weight harvested on day X, and FW0 is the fresh weight of the inoculum. The DW growth indices were used to calculate using this formula, Gi DW = [(DWx − DW0)/DW0] × 100%, where DWx is the amount of dry weight harvested on day X, and DW0 is the dry weight of the inoculum. The specific growth rate was calculated using this formula, μ = ln(X/X0)/Δt, where μ is the specific growth rate (1/d), X0 and X are the initial and final biomasses (g/L), and Δt is the culture time interval (d).

**Figure 2:**
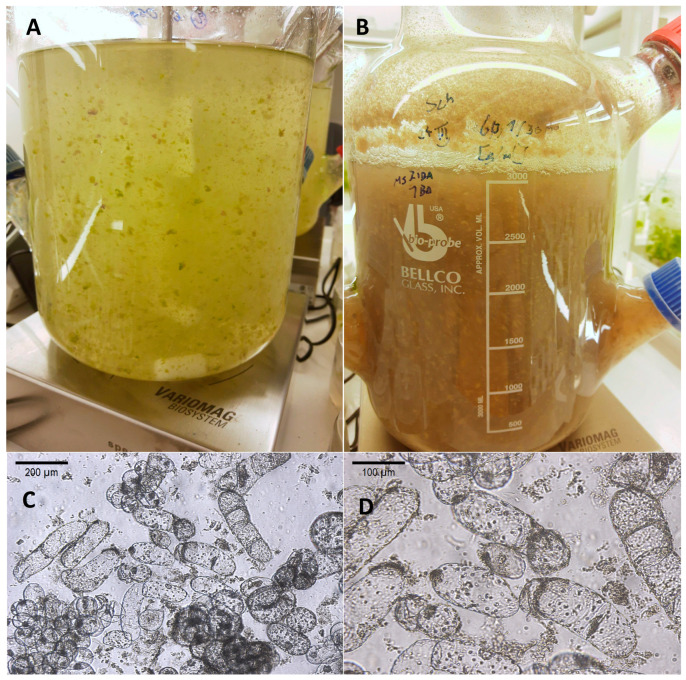
The suspension cell cultures of *S. henryi* grown in the stirred tank bioreactor: (**A**) general view, 5 day-old culture; (**B**) general view, 30-day-old culture; (**C**) microscope view, 100× magnification; (**D**) microscope view, 200× magnification.

The growth parameters of bioreactor-grown suspension cell cultures of *S. henryi* are presented in Figure 1. The maximum amount of fresh weight equal to ca. 50 g/L was recorded on days 20–30. These values corresponded to Gi values of ca. 220–250% and a dry weight content of 2.7–3.0 g/L in the respective time period. The established suspension culture consisted of biomass aggregates of different size (up to ca. 3 mm in diameter). In the course of the experiment, the color of the biomass changed gradually from yellow-green (day 5) to yellow-brown on day 30 (Figure 2). Microscope analysis revealed that the suspension culture consisted chiefly of cell aggregates of different sizes (Figure 2).

### 2.2. Lignan Profiling

Using the UHPLC-MS-MS method on the methanolic extract from the suspension cell cultures, eighteen lignans were identified, including fifteen dibenzocyclooctadiene lignans, one lignan from the aryltetralin group, and two neolignans (Table 1). The highest amounts were found for the neolignan, licarin A (3030 µg/100 g DW (dry weight), 15th day of cultivation), and the dibenzocyclooctadiene lignan rubrisandrin A (640 µg/100 g DW, 20th day of cultivation) (Table 1). The maximum total lignan content of 3700 µg/100 g DW (Table 1) was found for the extracts obtained from the suspension cell cultures that were cultured in a bioreactor for 15 days, suggesting that this is the most optimal culture duration for *S. henryi* suspension cell cultures.

The values are presented as mean values with standard deviations (±SD) and resulted from three independent experiments with three repetitions (n = 9). A significant difference is marked with a small letter a (*p* < 0.05).

### 2.3. Phenolic Compounds Profiling

The qualitative analysis of the polyphenolic compounds detected in the biomass extracts of the suspension cell cultures cultivated in a bioreactor confirmed the presence of polyphenolic compounds depending on the cultivation time (Table 2). The presence of 10 compounds in the extracts from the cultures cultivated over 5 days, 18 compounds after the 10-day culture cycle, 14 compounds after the 15-day culture cycle, 10 compounds after the 20-day culture cycle, 5 compounds after the 25-day culture cycle, and 12 compounds after 30-day breeding cycle was detected (Table 2). The molecules were classified as procyanidins and their derivatives. Their precursor, catechin, was detected as the dominant metabolite. Most compounds were identified or tentatively identified based on a comparison with the literature reports or available chemical standards. The parameters considered were the UV–VIS spectra, MS, and the MS^2^ profiles obtained for the major *m*/*z* signals recorded in negative ion mode.

The quantification of dominant compounds performed with the HPLC-DAD method for in vitro culture extracts was conducted for catechin (Table 3).

### 2.4. Inhibition of Enzymes Activity

#### 2.4.1. Tyrosinase Assay

**Figure 3:**
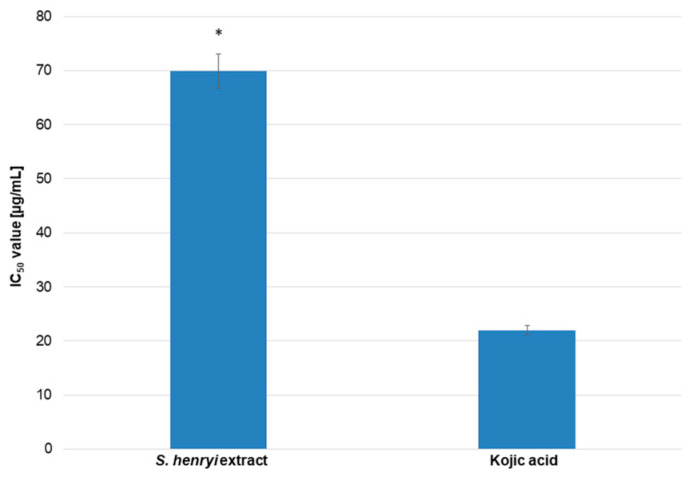
The *S. henryi* extracts from the suspension cultures grown in a bioreactor inhibited tyrosinase activity. The results (IC_50_ value [µg/mL]) are shown as the mean values with standard deviations (±SDs) obtained from three independent experiments with three repetitions (n = 9). Standard deviations are marked with error bars. A significant difference relative to kojic acid is marked with an asterisk “*” (*p* < 0.05).

The extract from *S. henryi* was investigated for its antityrosinase activity using spectrophotometric analysis [26]. In this experiment, kojic acid and L-DOPA were used as the standard compound and the substrate, respectively The obtained results showed that the *S. henryi* extract inhibited tyrosinase activity, and this effect was dose-dependent. The IC_50_ value was 69.92 µg/mL, and the thusactivity of the extract was only three times weaker than the standard, kojic acid, with an IC_50_ value of 21.99 µg/mL (Figure 3).

#### 2.4.2. Hyaluronidase Assay

**Figure 4:**
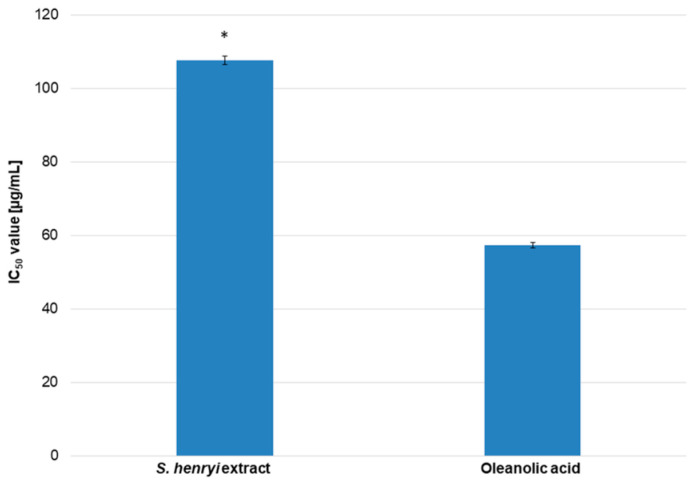
The effect of the *S. henryi* extracts from the suspension cultures grown in a bioreactor on hyaluronidase activity (expressed as IC_50_ value [µg/mL]). The results are presented as the mean values with standard deviations (±SDs) obtained from three experiments conducted independently with three repetitions (n = 9). The error bars indicate SDs. A significant difference relative to oleanolic acid is marked with an asterisk “*” (*p* < 0.05).

The antihyaluronidase activity of the *S. henryi* extract was estimated with spectrophotometry. In this experiment, hyaluronic and oleanolic acid were used as the substrate and the control, respectively. The results showed the dose-dependent effect of the *S. henryi* extract on enzyme activity. The activity of the extract was only two times weaker than the standard, oleanolic acid, with IC_50_ values of 107.63 µg/mL and 57.24 µg/mL, respectively (Figure 4).

### 2.5. Antioxidant Properties

The *S. henryi* extract was subjected to a series of antioxidant tests aimed at determining its ability to scavenge free radicals, reduce its capacity, and assess the impact on the potential protection of lipid membranes. The obtained IC_50_ values in comparison to those of the standard, ascorbic acid, are summarized in Table 4.

The obtained results showed that the *S. henryi* extracts exhibited a strong ability to reduce DPPH and ABTS radicals, achieving IC_50_ values at concentrations 21.3 and 17.91 µg/mL, respectively. The IC_50_ values for the reference substance were only slightly lower (Table 4. The Molybdenum reduction capability of the analyzed extract was higher than that of ascorbic acid (IC_50_ was 60.44 and 75.15 µg/mL, respectively), which confirmed the strong potential of the *S. henryi* extracts to reduce both the radicals and Mo (+6) to Mo (+5). The β-carotene bleaching assay indicated the dose-dependent capability of the *S. henryi* extract to protect the biological membranes, although the in vitro test indicated that its activity is almost ten times weaker than that of ascorbic acid (IC_50_ was 420.92 and 47.25 µg/mL, respectively).

The demonstrated results of the antioxidant tests performed for the *S. henryi* extracts indicated their strong ability to reduce the level of free radicals, similarly to ascorbic acid, as well as a weak capacity to protect lipids from oxidation. The obtained data showed that the *S. henryi* extracts have significant biological activities, which may be used in further in vivo studies, where pathological oxidative stress plays a key role.

### 2.6. Anticancer Potential In Vitro

**Figure 5:**
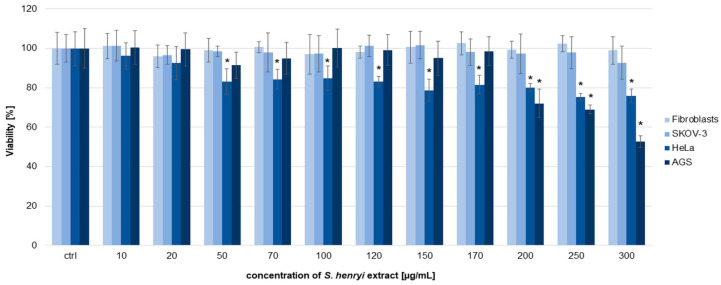
The viability of the cancer AGS, HeLa, and SKOV-3 cells and the non-cancer fibroblasts after 24 h of treatment with the *S. henryi* extracts from suspension cultures grown in a bioreactor. The control (ctrl) was the cells with DMSO (0.75% (*v*/*v*)). Two independent experiments were conducted, with six repetitions (n = 12) of the MTT assay. The results are shown as the mean values with standard deviations (±SDs, error bars). Significant differences relative to the control are marked with an asterisk “*” (*p* < 0.05).

To estimate the viability of cancer cells, SKOV-3, HeLa, and AGS and normal fibroblasts (as a non-cancer control), we treated the cells with the *S. henryi* extract for 24 h. The obtained results indicate that the extract did not show a significant cytotoxic effect on the tested cells in the used range of concentrations (Figure 5). The highest anticancer activity of the extract (among all the used cancer cell lines) was observed towards the AGS cell line; however, this effect occurred only above the extract concentration of 200 µg/mL. The viability of these cells decreased to 72.01%, 69.01%, and 52.72% at *S. henryi* extract concentrations of 200, 250, and 300 µg/mL, respectively. The effect of the extract on the HeLa cells was weak; however, this was observed above an extract concentration of 50 µg/mL. The viability of this cell line was 83.13%, 84.38%, 84.76%, 83.14%, 78.78%, 81.58%, 80.19%, 75.33%, and 75.89% for the extract concentrations of 50, 70, 100, 120, 150, 170, 200, 250, and 300 µg/mL, respectively. In the case of SKOV-3 cell and fibroblast viability, all the obtained values were above 90% for the used range of concentrations. In these experiments, we were not able to obtain IC_50_ values for the extract (at concentrations of 10–300 µg/mL), whereas oxaliplatin used as a positive control exhibited cytotoxic activity on the cells with IC_50_ of 17.90, 35.76, 102.16, and 95.79 µg/mL for the AGS, HeLa, and SKOV-3 cells and fibroblasts, respectively.

The extracts of *S. henryi* showed some anticancer potential, particularly on the cervical and gastric cancer cells, and therefore this study should be further evaluated with fractions and secondary metabolites obtained from the extracts.

## 3. Discussion

Plant in vitro cultures are increasingly becoming an alternative to obtaining bioactive metabolites compared to the in vivo growing of plants. The cultivation of plant cultures in vitro and biological activity tests on tissue extracts are becoming more and more popular [6,27,28,29]. The research described in this article is the first report on *S. henryi* suspension cell cultures cultivated in a bioreactor. Additionally, for the first time, the qualitative and quantitative analyses of compounds from the groups of lignans and polyphenols of *S. henryi* was carried out. The pioneering nature of research on a global scale also concerns the examination of the biological activity of the obtained extracts. These findings expand the knowledge about the chemical composition and biological activity of *S. henryi* plants.

The experiments demonstrated that establishing bioreactor cultures of *S. henryi* is feasible. However, some problems related to biomass viability and foaming were experienced during preliminary studies. The initial experiments were conducted using the bioreactor setup described earlier [30]. The system operated at a 3 L working volume, but without oxygen level monitoring employed before. At this stage, the established cultures exhibited strong foaming, which resulted in virtually the whole inocular biomass trapped in the foam and stuck to the bioreactor walls shortly after the experiment was started. As a consequence, the biomass decayed rapidly and stopped growing. In order to mitigate foam formation, the culture medium was supplemented with either silicone or fatty acid ester-type antifoam in concentrations up to 0.2% (*v*/*v*). The strategy proved to be unsuccessful as the above mentioned additives failed to prevent foaming and cell adhesion to the bioreactor walls in the long term. Both the antifoams were effective virtually only during the first 48 h of the experiment. As the growth cycle continued, foam eventually built up, clogging the bioreactor filters, and thus stopping its operation. Eventually, the foaming problem was solved by introducing a secondary stirrer plate, which was placed above the main stirrer plate and directly below the medium surface. The described approach based solely on the mechanical means of foam mitigation has previously been described by Junker [31]. The resulting bioreactor cultures of *S. henryi* proved to be viable; however, there was a rather long lag phase (up to the 10th day). It was followed by a phase of more intensive growth (days 10–20), after which the biomass concentration stabilized.

The UHPLC-MS/MS analysis of the bioreactor cell suspension extracts proved the presence of lignans from the dibenzocyclooctadiene (fifteen compounds), arylteralin (one compound), and neolignan (two compounds) groups (Table 1). The contents of individual compounds varied depending on the duration of the breeding cycle. The highest total lignan content was shown in the extracts grown in a 10-day breeding cycle, and the main compound was lycarin A. The obtained contents in the suspension extracts of *S. henryi* grown in a bioreactor with various types of lignan show that this species may have a therapeutic potential associated with the scientifically proven activities exhibited these compounds (e.g., hepatoprotective, anti-inflammatory, and anticancer). The obtained results encourage further research, which opens new possibilities for researching the use of extracts from suspension cell cultures as potential plant-based drugs.

The results of this phytochemical profiling study can be compared with those of a previous one carried out on another type of *S. henryi* culture, microshoots grown in PlantForm bioreactors [32]. Qualitative and quantitative determinations were conducted using the UHPLC-MS/MS method. Seventeen lignans from the dibenzocyclooctadiene, aryltetralin, and neolignan groups were identified, similarly to those in the suspension cell cultures in this study. The highest lignan amounts in the microshoot cultures were found for schisantherin B (361.24 mg/100 g DW), schisantherin A (61.65 mg/100 g DW), and 6-O-benzylgomisin O (68.53 mg/100 g DW), and they were 6.02, 2.06, and 1.7 times higher, respectively, in comparison to the present results. The dominant compound in the extracts from the suspension cell cultures was licarin A, the content of which was ca. 200 times higher than that in the extracts from microshoot cultures grown in PlantForm bioreactors.

In *S. henryi* agar microshoot and callus cultures, the profiling of dibenzocyclooctadiene lignans was performed using the HPLC-DAD method [12]. Their amounts ranged from 0.14 mg/100 g DW to 622.59 mg/100 g DW and from 0.20 mg/100 g DW to 17.49 mg/100 g, respectively.

Research on the determination of compounds from the group of polphenols in the extracts from *S. henryi* bioreactor-grown suspension cell cultures is pioneering. Qualitative analysis showed the presence of compounds mainly from the procyanidin group and its precursor, catechin (Table 2). The qualitative profiles of the extracts with different cultivation times differed in the number of estimated compounds. The presence of 10 compounds in the extracts from the cultures cultivated over 5th days, 18 compounds after the 10th day culture cycle, 14 compounds after the 15th day culture cycle, 10 compounds after the 20th day culture cycle, 5 compounds after the 25th day culture cycle, and 12 compounds after the 30th day breeding cycle was estimated. Procyanidins are compounds exhibiting valuable biological activities, e.g., anti-inflammatory, antioxidant, and anticancer properties, which could be used in pharmacy on a large scale [33,34,35].

The phenolic compounds have been estimated before in *S. henryi* microshoot and callus cultures grown on an agar medium, microshoots cultured in PlantForm bioreactors, as well as in suspensions and agitated microshoot cultures maintained in Erlenmayer flasks [36]. The qualitative assays of the suspension cultures confirmed eight compounds from the polyphenol group, mainly procyanidins, but also protocatechuic acid derivatives and catechin. Two compounds, protocatechuic acid (0.07 mg/100 g DW) and catechin (65.16 mg/100 g DW), were quantified. No protocatechuic acid derivatives were found in the extracts from the cultures grown in the bioreactor, but the presence of catechin was found as dominant. The catechin contents in the extracts from the suspension cell cultures grown in a 30-day culture cycle (65.16 mg/100 g DW) compared to those of the extracts from the suspension cell cultures grown in the bioreactor, were, respectively, 2.6 (5th day), 1.3 (10th day), 5.2 (15th day), 6 (20th day), 1.6 (25th day), and 3.7 (30th day) times lower depending on the duration of the cultivation period. Comparing the obtained results with the extracts from the other types of *S. henryi* in vitro culture, it can be concluded that the highest amounts of catechin are present in the extracts from the suspension cell cultures grown in the bioreactor (390.44 mg/100 g DW), which are higher by 17.3; 3.5; 5.9; and 3.1 times than those of the extracts from the agar callus cultures, as well as the agar-agitated and Plantform bioreactor-grown microshoots.

In the *S. henryi* agar microshoot and callus cultures, the polyphenol profile determined using the HPLC-DAD method showed the presence of seven phenolic acids (gallic, neochlorogenic, caftaric, chlorogenic, caffeic, syringic, and vanillic) and six flavonoids (3-quercetin galactoside, 3-quercetin glucoramnoside, kaempferol 3-galactoside, quercetin-3-rhamnoside, quercetin, and kaempferol) [12]. The above-mentioned compounds were not found in the suspension extracts grown in the bioreactor in the present study. In the previous studies, the presence of catechin and coumaroylquinic acid was not detected [12].

Szopa et al. [37], based on research on another species from the Schisandraceae family, *S. rubriflora*, found 27 compounds from phenolic acids and flavonoids (e.g., neochlorogenic acid, chlorogenic acid, cryptochlorogenic acid, hyperoside, rutoside, isoquercitrin, guaijaverin, trifolin, quercetin, kaempferol, and isorhamnetin). No compound overlaps with the compounds detected in the extracts from the suspension cell cultures cultivated in the bioreactor. In the suspension cultures, the procyanidin compounds were mainly synthesized.

For the pharmacopoeial species of the *Schisandra* genus, *S. chinensis* microshoot cultures were carried out in large-scale biotechnological experiments in various types of bioreactor, bubble-column bioreactors with biomass immobilization, balloon-type bioreactors, gas-phase spray bioreactors, and RITA^®^ and PlantForm temporary immersion systems over 30th- and 60th-day growth periods [38]. The phenolic profile of the extracts was also different from that of the suspension cell cultures of *S. henryi*.

The suspension cell cultures of another species, *Verbena officinalis*, were tested for phenolic compound production by Kubica et al. [7]. The experiments conducted on callus suspensions, as well as bioreactor-grown (balloon and stirred tank bioreactors) cultures, showed the presence of six phenolic acids (protocatechuic, chlorogenic, vanillic, caffeic, ferulic, and rosmarinic). It was found that a high phenolic content was found in the extracts from the suspension cell cultures.

Suspension cell cultures of *Phoenix dactylifera* were studied for catechin production [39]. The highest catechin content (26.6 µg/g DW), as well as other phenolic compounds, caffeic acid (31.4 µg/g DW) and kaempferol (13.6 µg/g DW), were indicated in the cell extracts after elicitation with 50 mg/L of salicylic acid. The obtained catechin content was 1.46 times lower compared to that of the *S. henryi* suspension culture.

The catechin content was also studied in suspension cell cultures of *Camellia sinensis* [40]. The content was equal 12.13 µg/mL, which was over 300 times lower than that for the *S. henryi* cell suspension cultures.

The ability of the *S. henryi* extract to inhibit the activity of tyrosinase and hyaluronidase enzymes was under revision in this study for the first time (Figure 3 and Figure 4). The aim of this research was to obtain such a concentration of the extract to inhibit enzyme activity by 50% (IC_50_). The results indicated the dose-dependent capability to inhibit both the enzymes in a slightly weaker manner than that of the standards.

The obtained data showed that the *S. henryi* extract is a potential reducer of oxidative stress among the skin layers (Table 4). Both antiradical and antityrosinase activities may reduce the free radicals generated during the activation of tyrosinase, as well as radicals sourced from internal and external factors. The tested extract may be capable of inhibiting the formation of melanin and reducing oxidative stress associated with its formation. Additionally, by inhibiting the activity of hyaluronidase in vitro, the ability of the extract to reduce the decomposition of hyaluronic acid was proven. Hyaluronidase activity is also associated with inflammation. The skin, as the largest and most external organ of the body, is, at the same time, the most exposed to external free radicals and related inflammation. The tested extract, therefore, seems to be a good candidate for cosmetic formulations aimed at improving hydration and reducing the formation of discoloration and inflammatory processes in the skin.

The study on the cytotoxic activity of the *S. henryi* extract revealed its anticancer potential, especially against gastric and cervical cancer cells (Figure 5). This effect may be due to the presence of many different metabolites in the extract. Wang et al. showed that one of the main plant metabolites, schisantherin A, inhibits the proliferation of the human gastric cancer cell lines SGC-7901 and MKN45 [41]. In turn, procyanidin B2 induces apoptosis and autophagy in human gastric cancer cells BGC-823 and SGC-7901 [42]. Catechin can also effectively inhibit the migration and proliferation of the human gastric cell lines MGC-803 and SGC-7901 [43]. However, further studies in this field need to be carried out with *S. henryi* isolated fractions and metabolites.

## 4. Materials and Methods

### 4.1. In Vitro Callus Culture

Plant material for initiating in vitro cultures of *S. henryi* was obtained from the “Clematis -Źródło Dobrych Pnączy” company (Clematis Spółka z o.o., Duchanicka 27 street, 05-800, Pruszków, Poland, 52.19847149412482, 20.7924781961298; www.e-clematis.com). The plant species was identified by Dr. Szczepan Marczyński (head of the Clematis arboretum) and Prof. Agnieszka Szopa. The cultures were initiated from leaf buds collected in April 2018. The leaf buds were washed with 70% ethanol (30 s), and then subjected to further sterilization with 0.2% HgCl_2_ (mercuric chloride II) for 6 min. The sterile buds were rinsed with sterile redefined water and transferred to medium according to Murashige and Skoog (MS) [44], with 30 g/L of saccharose, 7.2 g/L agar (Duchefa Biochemie, Haarlem, Netherlands), and the following plant growth regulators (PGRs): cytokinin, 1 mg/L BA (6-benzyladenine, Sigma-Aldrich, Saint Louis, MI, USA); and auxin, 0.5 mg/L NAA (1-naphthaleneacetic acid, Sigma-Aldrich, Saint Louis, MI, USA).

Callus tissue appeared around initial shoots. The callus tissue was separated and multiplied for experimental purposes on agar-solidified MS medium with 1 mg/L BA and 1 mg/L NAA according to above described protocol every 30 days for 5 passages [44].

### 4.2. Experimental Suspension Cell Cultures Grown in Bioreactor

**Figure 6:**
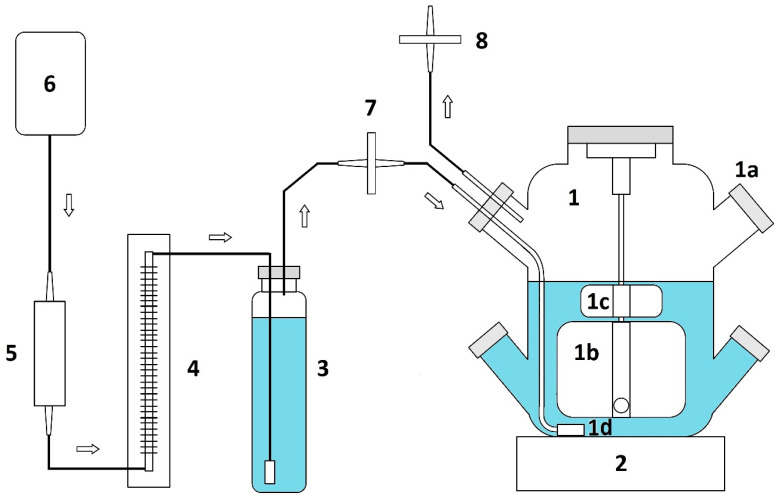
A schematic diagram of the stirred tank bioreactor employed in this study: 1 the culture, vessel, 1a the inoculation port, 1b main plate impeller, 1c the secondary plate impeller, 1d the glass frit sparger; 2 the magnetic stirrer; 3 the air humidifier; 4 the flow meter; 5 the polypropylene wool prefilter; 6 the air pump; 7 and 8 the air sterilization filters. The arrows indicate the direction of air flow.

A stirred tank bioreactor of the spinner flask type equipped with a magnetically driven plate stirrer was used. Preliminary experiments were conducted using the bioreactor system described earlier [30], albeit without controlling the dissolved oxygen levels. Eventually, the system was modified by placing an additional stirrer above the main stirrer plate. The bioreactor configuration used in this research is depicted in Figure 6. The working volume was set at 3 L, and the inoculum was 20 g/L of medium. The *S. henryi* cultures were propagated using Murashige and Skoog (MS) medium with the addition of 3% (*w*/*v*) sucrose and 2 mg/L indolyl-3-butyric acid (IBA) and 1 mg/L 6-benzyladenine (BA). Each time, the callus biomass grown on stationary MS medium for about 20 days was used to initiate the experiment. The callus required for cultivation was collected using a metal spoon and weighed in a glass beaker until the mass was 60 g (finally corresponding to 20 g/L of medium). Then, the inoculum was suspended in the medium (approx. 50 mL), and the callus agglomerates were crushed using a glass piston. The above activities were performed under aseptic conditions. The materials prepared in this way were placed in a culture vessel with a growth medium (3 L). The tightly closed bioreactor was placed on a magnetic stirrer (Variomag Biosystem Direct magnetic stirrer, Thermo Fisher Scientific, Waltham, MA, USA, mixing speed 40 rpm) and connected to an aeration system (Optima air pump, Hagen, Montreal, Canada, 0.5 L/min, 0.17 vvm). The biomass was collected at 5-day intervals, starting from day 5 to day 30, each time the installation was emptied (batch culture). The experiments in the bioreactor were conducted with four repetitions (n = 4).

### 4.3. Extraction

The methanol extracts were prepared from the cell suspension culture biomass cultivated in the bioreactor. Samples (0.3 g, 3 replicates) were extracted twice with 6 mL of methanol (HPLC grade, Merck, Darmstadt, Germany) in an ultrasonic bath (Sonic 2, POLSONIC, Warsaw, Poland) for 30 min each time. The obtained extracts were then centrifuged for 8 min at 4000 rpm (MPW-223E, MPW, Warsaw, Poland). The collected extracts were filtered using a Millex^®^ GP, 0.22 µm, Filter Unit (Millipore, Bedford, MA, USA).

### 4.4. Lignan Profiling with UHPLC-MS/MS Method

The quantitative analysis of lignans was performed using a UHPLC-MS/MS tandem mass spectrometer with a triple quadrupole mass filter (QQQ) (Agilent 6410 LC/MS, CA, USA) connected to an ultra-high-performance chromatograph (Agilent 1260, CA, USA) as detailed previously [45]. Separation was achieved with a Kinetex C18 analytical column (150 × 4.6 mm, 2.6 μm) using a gradient of 50% methanol (A) vs. 100% methanol (B) both with 0.1% formic acid (ramped from 20% to 65% B over 22 min at a flow rate of 0.5 mL/min, a column temperature of 60 °C, and an injection volume of 2 μL). Analyses were carried out in positive ionization mode (+ESI) using selected ion transitions (MRM mode). Identification and quantification were performed by comparing the lignans with pure standards obtained from ChemFaces Biochemical Co., Ltd. (Wuhan, China). The quantitated lignans included wulignan A1, rubrisandrin A, interiotherin C (rubriflorin A), schisandrin A, gomisin D, gomisin J, gomisin, gomisin G, licarin B, epigomisin O, gomisin O, meso-dihydroguaiaretic acid, schisantherin A, schisantherin B, licarin A, schisanhenol, deoxyschisandrin, fragransin A, pregomisin, gomisin N, 6-O-benzylgomisin O, and schisandrin C; and for the leaf extracts, this included schisandrin, gomisin A, gomisin G, schisantherin A, schisantherin B, schisanhenol, deoxyschisandrin, γ-schisandrin, and schisandrin C. Further technical details are provided by Szopa et al. [45], as well as in the Supplementary Materials. The results are expressed in µg/100 g DW (dry weight).

### 4.5. Phenolic Compound Profiling with UHPLC-DAD-ESI-MS^3^ and HPLC–DAD Methods

UHPLC-DAD-ESI-MS^3^ analysis was achieved using a UHPLC-3000 RS system (Dionex, Leipzig, Germany) equipped with a DAD detector and splitless connection with an AmaZon SL ion trap mass spectrometer with an ESI interface (Bruker Daltonik GmbH, Bremen, Germany). The UV spectra were obtained over the range of 200–450 nm. The parameters of the MS unit were as follows: nebulizer pressure 40 psi, drying gas flow rate 9 L/min, nitrogen gas temperature 134 °C, and capillary voltage 4.5 kV. The mass spectra were registered by scanning from *m*/*z* 70 to 2200. Fragmentation was performed using SmarFrag mode with an amplitude between 0.6 to 3.0 V for all the detected ions. At the same time, the most abundant precursor ions in the MS spectrum were subjected to fragmentation. Kinetex XB-C_18_ chromatography columns were used (Phenomenex, Torrance, CA, USA, 150 mm × 3.0 mm × 2.6 µm for the phytochemical analysis of the control sample; 150 mm × 2.1 mm × 1.7 µm for batch culture extract analysis). The mobile phase (A) was H_2_O/formic acid (100:0.1, *v*/*v*), and the mobile phase (B) was acetonitrile/formic acid (100:0.1, *v*/*v*). All the solvents were of analytical or LC–MS grade. The gradient program and the flow rate were 0–20 min 1–26% B, 20–80 min 26–75% B, 80–90 min 75–100% B, and 0.3 mL/min, respectively. The column oven temperature was set to 25 °C. The injection volume was 3 µL.

HPLC–DAD analysis was performed to quantify the detected compounds. Analysis was performed using the validated method [12] of using a Merck Hitachi liquid chromatograph (LaChrom Elite, Darmstadt, Germany) with a DAD L-2455 detector. Separation was performed on a Purospher RP-18 column (250 × 4 mm; 5 μm, Merck, Germany). The mobile phase consisted of A-methanol, 0.5% acetic acid 1:4, and B-methanol (*v*/*v*). The flow rate was 1 mL/min at 25 °C. The gradient was 100% A for 0–20 min, 100–80% A for 20–35 min, 80–70% A and 20–30% B for 35–45 min, 70–60% A and 30–40% B for 45–55 min, 60–50% A and 40–50% B for 55–60 min, 50–25% A, and 50–75% B for 60–65 min, 25–0% A and 75–100% B for 65–70 min, 0–0% A and 100–100% B for 70–75 min, 0–100% A, and 100–0% B for 75–80 min, and 100–100% A, and 0–0% B for 80–90 min. The injection volume was 10 μL. The UV spectra were captured over the range of 200–400 nm; the compounds were identified at 254 nm. The identification and quantification of catechin were accomplished by comparing the retention times, the UV spectra with a standard substance (Sigma Aldrich, Saint Louis, MI, USA), and the verified fragmentation spectra by UHPLC-DAD-ESI-MS^3^. Quantification was performed using the calibration curve method. The results are expressed in mg/100 g DW (dry weight).

### 4.6. Chemical Reagents for Biological Activity Tests

DPPH (2,2-diphenyl-1-picrylhydrazyl), ABTS (2,2′-azino-bis(3-ethylbenzothiazoline-6-sulfonic acid)) diammonium salt, ascorbic acid, kojic acid, oleanolic acid, potassium persulfate, bovine serum albumin (BSA), hyaluronic acid, hyaluronidase from bovine testes type I-S, DMSO (dimethyl sulfoxide), sodium phosphate buffer (pH 7), tyrosinase (tyrosinase from mushroom), Tween-20, L-DOPA, linoleic acid, phosphate buffer (0.175 mM, pH 6.8), and β-carotene were obtained from Merck Millipore (Burlington, MA, USA). HPLC-grade methanol, TRIS-HCl (0.2 M, pH 8), chloroform, ammonium molybdate, and sulfuric acid were purchased from P.O.Ch. (Gliwice, Poland).

### 4.7. Hyaluronidase Assay

The assay was performed with spectrophotometry [46]. Hyaluronidase (100 U/mL), sodium phosphate buffer (20 mM, pH 7.0; with 77 mM NaCl and BSA 0.01%), and the analyzed extract were components of the mixture. The standard was oleanolic acid. Hyaluronic acid (HA) was added to the mixture and incubated at 37 °C for 45 min. Acid albumin solution was used in precipitation of the undigested HA. The results were obtained at λ = 600 nm (Epoch, BioTek System, Winooski, VT, USA).

The percentage of inhibition was calculated as Hyaluronidase activity = 100% − [ΔA Sample/ΔA Positive Control].

### 4.8. Tyrosinase Assay

The test was performed with spectrophotometry [26]. L-DOPA and kojic acid were used as the substrate and the standard compound, respectively. The enzyme tyrosinase (120 U), phosphate buffer (0.175 mM, pH 6.8), and the analyzed extract were used in the reaction mixture. The reaction was started by adding L-DOPA. The results were recorded at λ = 475 nm every 20 s for 20 min (Epoch BioTek System, Winooski, VT, USA). L-DOPA, the enzyme, and phosphate buffer were the control.

Tyrosinase inhibition was calculated according to the following equation:

Tyrosinase inhibition (%) = [(A_control_ − A_sample_)/A_control_] × 100% (1)

### 4.9. Antioxidant Properties

#### 4.9.1. DPPH Assay

This assay was conducted with the *S. henryi* extract and ascorbic acid as the standard. The results were obtained with spectrophotometry (λ = 517 nm, a microplate reader (Epoch, BioTek System, Winooski, VT, USA)) [47]. DPPH methanolic solution (50 μL of 0.06 mM) and the extract at different concentrations were in the reaction mixture. DPPH and water were the control.

DPPH inhibition was calculated according to the following equation:

DPPH inhibition (%) = [(A_control_ − A_sample_)/A_control_] × 100% (2)

#### 4.9.2. ABTS Assay

The ABTS assay with the *S. henryi* extract was conducted with ascorbic acid as the standard compound and spectrophotometry (λ = 750 nm, a microplate reader (Epoch, BioTek System, Winooski, VT, USA)) [48]. The extract dissolved in water at different concentrations mixed with ABTS solution (170 μL, 3.5 mM potassium persulfate, 2 mM ABTS diammonium salt) and water. The control was ABTS solution and water.

ABTS inhibition was calculated according to the following equation:

ABTS inhibition (%) = [(A_control_ − A_sample_)/A_control_] × 100% (3)

#### 4.9.3. Reduction-of-Mo(+6)-to-Mo(+5) Assay

This test was performed spectrophotometrically (λ = 695 nm, a microplate reader (Epoch, BioTek System, Winooski, VT, USA)), with ascorbic acid as the standard compound [49]. The reaction mixture (28 mM sodium phosphate, 0.6 M sulfuric acid, and 4 mM ammonium molybdate) was added to the extract dilutions and incubated (95 °C, 90 min). The reaction mixture and water were the control.

The standard curve for ascorbic acid (0.01–1 mg/mL) was used for calculating the percentage of reduced Molybdenum ions.

#### 4.9.4. βCarotene Bleaching Assay

In the assay, ascorbic acid was used as a standard. The extract was added to the reaction solution (prepared according to [37,50]), shaken, and incubated (120 min, 50 °C). Then, the results were obtained spectrophotometrically (at 470 nm, in t_0_ and t_2h_). Water and the reaction solution were used as the control.

The activity of the *S. henryi* extract was calculated with the following equation:

Antioxidant activity (%)= [1−((*A*_0*s*_−*A*_120*s*_)/(*A*_0*c*_−*A*_120*c*_))] × 100% (4)

where *A*_0*s*_—absorbance of the sample, time 0 min; *A*_120*s*_—absorbance of the sample, after 120 min; *A*_0*c*_—absorbance of the control, time 0 min; *A*_120*c*_—absorbance of the control, after 120 min.

### 4.10. Anticancer Studies

#### 4.10.1. Cell Culture

Human gastric cancer AGS, ovarian cancer SKOV-3, and cervical adenocarcinoma HeLa S3 cell lines were obtained from the American Type Culture Collection (ATCC, Manassas, VA, USA). Primary human fibroblasts were purchased from the LGC Standards (Teddington, Middlesex, UK). Fibroblast Growth Medium with Supplement Mix, Dulbecco’s Modified Eagle’s Medium (DMEM), McCoy’s Medium, and DMEM/Ham’s F-12 were used for the fibroblasts and the HeLa S3, SKOV-3, and AGS cell cultures (Merck Millipore, Burlington, MA, USA), respectively. Supplements (100 mg/mL of streptomycin, 100 units/mL of penicillin, and 10% (*v*/*v*) fetal bovine serum (FBS)) were added to all the media. The cells were incubated at 37 °C and 5% CO_2_.

#### 4.10.2. MTT [3-(4,5-Dimethylthiazol-2-yl)-2,5-Diphenyltetrazolium Bromide] Assay

An MTT assay was used to assess the viability of all the tested cell lines after treatment with the *S. henryi* extract [51,52]. The cells were seeded at a density of 5 × 10^3^ cells/well and treated with the extract dissolved in DMSO (10–300 µg/mL). The maximum concentration of the extract’s solvent in the media did not exceed 0.75% (*v*/*v*). The positive control was oxaliplatin (at concentrations of 20–400 µg/mL). Formazan crystals were dissolved in DMSO, and the absorbance of the solution was measured with a microtiter plate reader (Epoch, BioTek Instruments, Winooski, VT, USA). GraFit software v.7 (Erithacus Software, East Grinstead, West Sussex, UK) was used to analyze the obtained results.

### 4.11. Statistical Analysis

The STATISTICA 12.0 software package (StatSoft. Inc., Tulsa, OK, USA) was used in statistical analysis. All the data are expressed as mean values ± standard deviation (±SD). For the comparison of the obtained results with the control samples, the Student’s *t*-test was performed. Statistical significance was set at *p* < 0.05.

## Figures and Tables

**Table 1:** Contents (µg/100 g DW ± SD) of lignans in *S. henryi* extracts from suspension cell cultures grown in bioreactor.

Compound	Day of Cultivation						
	0 th	5 th	10 th	15 th	20 th	25 th	30 th
Wulignan A1	80 ± 0.01 ^a^	0.27 ± 0.09 ^b^	310 ± 0.23 ^d^	0.09 ± 0.02 ^b^	1101 ± 0.01 ^c^	120 ± 0.08 ^e^	100 ± 0.04 ^a^
Rubrisandrin A	90 ± 0.01 ^a^	150 ± 0.04 ^e^	160 ± 0.09 ^e^	360 ± 0.13 ^d^	640 ± 0.26 ^f^	310 ± 0.18 ^d^	350 ± 0.22 ^d^
Interiotherin C (Rubriflorin A)	20 ± 0.01 ^g^	20 ± 0.01 ^g^	10 ± 0.01 ^g^	0.02 ± 0.01	10 ± 0.01 ^g^	10 ± 0.01 ^g^	10 ± 0.01 ^g^
Schisandrin (Schisandrol A)	nd	10 ± 0.01 ^g^	10 ± 0.01 ^g^	10 ± 0.01 ^g^	20 ± 0.01 ^g^	10 ± 0.01 ^g^	70 ± 0.01 ^a^
Gomisin D	10 ± 0.01 ^g^	nd	10 ± 0.01 ^g^	30 ± 0.01 ^h^	40 ± 0.01 ^h^	20 ± 0.01 ^g^	10 ± 0.01 ^g^
Gomisin J	nd	10 ± 0.01 ^g^	20 ± 0.01 ^g^	10 ± 0.01 ^g^	10 ± 0.01 ^g^	10 ± 0.01 ^g^	10 ± 0.01 ^g^
Gomisin A	20 ± 0.01 ^g^	10 ± 0.01 ^g^	50 ± 0.01 ^h^	nd	nd	nd	40 ± 0.01 ^h^
Licarin B	nd	10 ± 0.01 ^g^	nd	10 ± 0.01 ^g^	nd	nd	nd
Epigomisin O	nd	nd	20 ± 0.01 ^g^	nd	nd	nd	10 ± 0.01 ^g^
Gomisin O	nd	20 ± 0.01 ^g^	20 ± 0.01 ^g^	70 ± 0.01 ^h^	10 ± 0.01 ^g^	nd	50 ± 0.01 ^h^
Schisantherin A	nd	30 ± 0.01 ^g^	nd	10 ± 0.01 ^g^	nd	nd	nd
Schisantherin B	20 ± 0.01 ^g^	60 ± 0.01 ^h^	20 ± 0.01 ^g^	20 ± 0.01 ^g^	nd	nd	nd
Licarin A	1230 ± 0.97 ^c^	5820 ± 1.34 ^i^	1216 ± 2.67 ^c^	3030 ± 0.79 ^j^	1140 ± 0.56 ^c^	2730 ± 1.03 ^k^	540 ± 0.24 ^f^
Schisanhenol	nd	10 ± 0.01 ^g^	20 ± 0.01 ^g^	nd	nd	10 ± 0.01 ^g^	nd
Schisandrin A (Deoxyschisandrin)	20 ± 0.01 ^g^	40 ± 0.01 ^g^	30 ± 0.01 ^g^	20 ± 0.01 ^g^	nd	nd	nd
Fragransin A	nd	nd	10 ± 0.01 ^g^	nd	nd	nd	nd
Gomisin N	nd	10 ± 0.01 ^g^	10 ± 0.01 ^g^	20 ± 0.01 ^g^	nd	10 ± 0.01 ^g^	nd
6-O-Benzylgomisin O	nd	20 ± 0.01 ^g^	30 ± 0.01 ^g^	20 ± 0.01 ^g^	nd	40 ± 0.01 ^h^	20 ± 0.01 ^g^
Total content	1490 ± 0.97 ^a^	6490 ± 1.47 ^b^	1289 ± 2.99 ^a^	3700 ± 0.94 ^c^	1980 ±0.83 ^d^	3270 ± 1.29 ^e^	1270 ± 0.5 ^a^

nd—not detected.

**Table 2:** Phenolic compound profiling of *S. henryi* extracts from suspension cultures grown in bioreactor; b—base peak.

Compound	Retention Time (min)	UV-Vis	Precursor Ion (*m*/*z*)	Pseudomolecular Ion	MS^2^ Fragments
Procyanidin trimer type C isomer ^1,2,3,4,6^	9.2	<210, 252, 287	865	[M-H]^−^	287, 363, 407, 449, 543, 695b, 739
Procyanidin dimer type A isomer ^2^	10.8	<210, 278	575	[M-H]^−^	171B167, 257, 287, 289, 361, 407b, 423, 449,499, 557
Procyanidin dimer type A isomer ^2^	11.7	<210, 278	575	[M-H]^−^	167, 257, 287, 289, 361, 407b, 423, 449,499, 557
Procyanidin dimer type B isomer ^1,2,3,4,5,6^	12.3	<210, 279	577	[M-H]^−^	245, 289, 407, 425, 451, 559
Procyanidin dimer type B isomer ^2,4,5,6^	12.7	<210, 278	577	[M-H]*^−^*	245, 289, 407, 425, 451, 559
Catechin ^1,2,3,4,5,6^	13.2	<210, 279	289	[M-H]*^−^*	205, 245b
Procyanidin trimer type C isomer ^1,2,4,5,6^	13.9	<210, 278	865	[M-H]*^−^*	287, 363, 407, 449, 543, 695b, 739
Procyanidin dimer type B isomer ^2,3^	13.9	<210, 278	577	[M-H]*^−^*	245, 289, 407, 425, 451, 559
Procyanidin dimer type B isomer ^2^	14.2	<210, 279	577	[M-H]*^−^*	245, 289, 407, 425, 451, 559
Procyanidin trimer type C isomer ^2,6^	14.2	<210, 279	865	[M-H]*^−^*	287, 363, 407, 449, 543, 695b, 739
Procyanidin tetramer isomer ^1,3,4,5,6^	14.3	<210, 275	1153	[M-H]*^−^*	289, 344, 407, 413, 452, 575, 577, 695, 739, 866, 984b
Procyanidin tetramer isomer ^1,2,3,6^	14.9	<210, 278	1153	[M-H]*^−^*	289, 344, 407, 413, 452, 575, 577, 695, 739, 866, 984b
Procyanidin tetramer isomer ^4^	15	<210, 278	1153	[M-H]*^−^*	289, 344, 407, 413, 452, 575, 577, 695, 739, 866, 984b
Coumaroylquinic acid ^1^	15.2	<210, 285, 310	337	[M-H]^−^	163, 191b
Coumaroylquinic acid ^2,3,6^	15.3	<210, 277, 310	337	[M-H]*^−^*	163, 163b, 191b
BProcyanidin tetramer isomer ^3^	B15.3	<210, 278, 311	1153	[M-H]*^−^*	289, 344, 407, 413, 452, 575, 577, 695, 739, 866, 984b
Procyanidin dimer type B isomer ^1,2,3,4,6^	15.6	<210, 278	577	[M-H]*^−^*	245, 289, 407, 425, 451, 559
Procyanidin trimer type C isomer ^1,2,3,4,6^	15.6	<210, 278	865	[M-H]*^−^*	287, 363, 407, 449, 543, 695b, 739
Procyanidin trimer type C isomer ^2^	16	<210, 275	865	[M-H]*^−^*	287, 363, 407, 449, 543, 695b, 739
Unknown hexoside ^2^	16.3	<210, 279	391	[M+HCOOH-H]*^−^*	161, 183b, 207, 255, 345
Procyanidin tetramer isomer ^2,3^	16.7	<210, 279	1153	[M-H]*^−^*	289, 344, 407, 413, 452, 575, 577, 695, 739, 866, 984b
Coumaroylquinic acid ^2^	16.9	<210, 279, 314	337	[M-H]*^−^*	163, 191b
Procyanidin trimer type C isomer ^4,6^	17.2	<210, 279	865	[M-H]*^−^*	287, 363, 407, 449, 543, 695b, 739
Procyanidin tetramer isomer ^2,3^	17.2	<20, 277	1153	[M-H]*^−^*	289, 344, 407, 413, 452, 575, 577, 695, 739, 866, 984b
Procyanidin trimer type C isomer ^6^	17.6	<215, 278	865	[M-H]^−^	287, 363, 407, 449, 543, 695b, 739

The presence of compounds in the biomass extracts in the cultures cultivated in a bioreactor depending on the duration of the growth period: ^1^—5th day; ^2^—10th day; ^3^—15th day; ^4^—20th day; ^5^—25th day; ^6^—30th day.

**Table 3:** The quantitative analysis (mg/100 g DW ± SD) of catechin in the *S. henryi* extracts from the suspension cultures grown in a bioreactor.

Compound	Day of Cultivation					
	5th	10th	15th	20th	25th	30th
Catechin	169.60 ± 0.10	84.62 ± 0.05	337.45 ± 0.49	390.44 ± 0.61	107.08 ± 0.34	240.26 ± 0.61

**Table 4:** Antioxidant properties of *S. henryi* extracts from suspension cultures grown in bioreactor presented as IC_50_ values [µg/mL].

Assay	IC_50_ [µg/mL]	
	*S. henryi* Extract	Ascorbic Acid
DPPH	21.30 ± 0.90 *	11.07 ± 0.23
ABTS	17.91 ± 0.78 *	15.05 ± 0.19
Molybdenum reduction	60.44 ± 0.24 *	75.15 ± 1.23
β-Carotene bleaching	420.92 ± 4.2 *	47.25 ± 0.79

IC_50_ values with standard deviations (±SDs) resulted from three experiments independently performed with three repetitions (n = 9). All obtained results for *S. henryi* extracts are significantly different (*) from values of standard, ascorbic acid (Student’s *t*-test, *p* < 0.05).

## Data Availability

The authors confirm that the data supporting the findings of this study are available within this article.

## Supplementary Materials

The supporting information can be downloaded at: https://www.mdpi.com/article/10.3390/molecules29225260/s1, Figure S1: Mass spectra of lignan standards in full scan mode. Ionization conditions applied: positive ionization (+ESI), drying gas temperature—350 °C, gas flow—12 L/min, nebulizer pressure—35 psi; Figure S2: Chromatogram of standard mixture; Figure S3: Calibration linearity functions for monitored compounds; Table S1: Monitored lignans and selected experimental parameters.
