# Supplementary material for: Phytochemical Profiling and Biological Activities of Extracts from Bioreactor-Grown Suspension Cell Cultures of Schisandra henryi
Source: Molecules. 2024 Nov 7;29(22):5260. doi: 10.3390/molecules29225260 (PMC11596403; doi:10.3390/molecules29225260)
Supplement: Supplementary file 1 [file molecules-29-05260-s001.zip › molecules-3259360-supplementary.pdf]

Supplementary materials

Phytochemical Profiling and Biological Activities of Extracts from Bioreactor-Grown Suspension Cell Cultures of *Schisandra henryi*

Karolina Jafernik [1], Adam Kokotkiewicz [2], Michał Dziurka [3], Aleksandra Kruk [4], Anna Hering [5], Krzysztof Jędrzejewski [2], Piotr Waligórski [3], Piotr Graczyk [5], Paweł Kubica [1], Justyna Stefanowicz-Hajduk [5], Sebastian Granica [4], Maria Łuczkiewicz [2] and Agnieszka Szopa [1,*]

[1] Department of Medicinal Plant and Mushroom Biotechnology, Jagiellonian University, Medical College, ul. Medyczna 9, 30-688 Kraków, Poland; karolina.jafernik@doctoral.uj.edu.pl (K.J.); p.kubica@uj.edu.pl (P.K.)
[2] Department of Pharmacognosy, Medical University of Gdańsk, Al. Generała Józefa Hallera 107, 80-416 Gdańsk, Poland; adam.kokotkiewicz@gumed.edu.pl (A.K.); jedrzejewski.k.j@gumed.edu.pl (K.J.); maria.luczkiewicz@gumed.edu.pl (M.Ł.)
[3] Polish Academy of Sciences, The Franciszek Górski Institute of Plant Physiology, ul. Niezapominajek 21, 30-239 Kraków, Poland; michal.dziurka@gmail.com (M.D.); p.waligorski@ifr-pan.edu.pl (P.W.)
[4] Microbiota Lab, Department of Pharmaceutical Biology, Medical University of Warsaw, ul. Banacha 1, 02-097 Warszawa, Poland; aleksandra.kruk@wum.edu.pl (A.K.); sebastian.granica@wum.edu.pl (S.G.)
[5] Department of Biology and Pharmaceutical Botany, Faculty of Pharmacy, Medical University of Gdańsk, Al. Generała Józefa Hallera 107, 80-416 Gdańsk, Poland; anna.hering@gumed.edu.pl (A.H.); piotr.graczyk@gumed.edu.pl (P.G.); justyna.stefanowicz-hajduk@gumed.edu.pl (J.S.-H.)

* Correspondence: a.szopa@uj.edu.pl

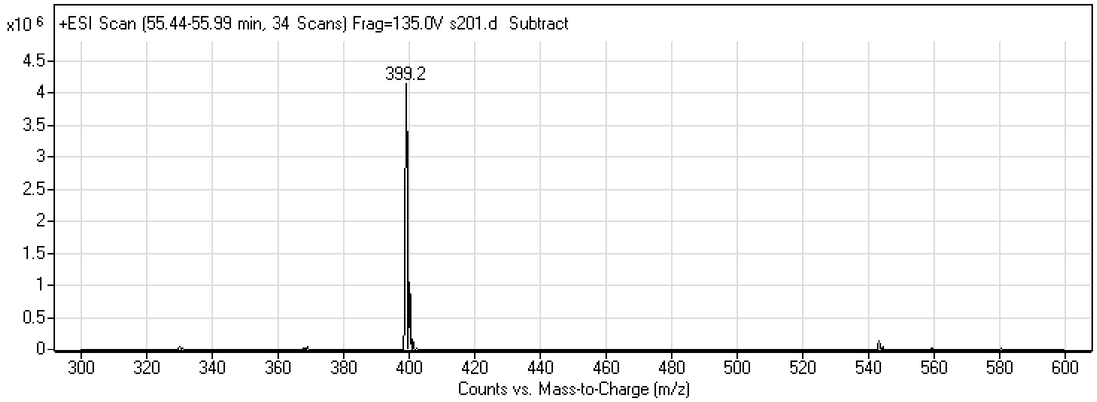

S01

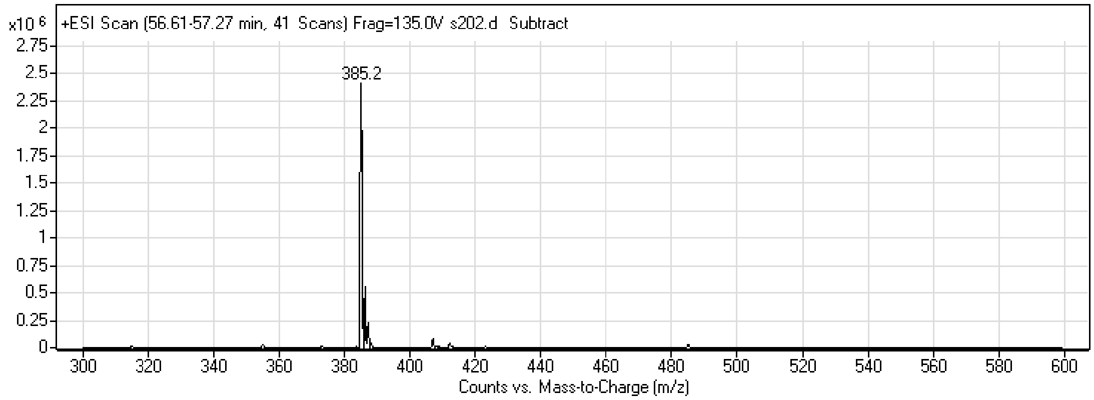

S02

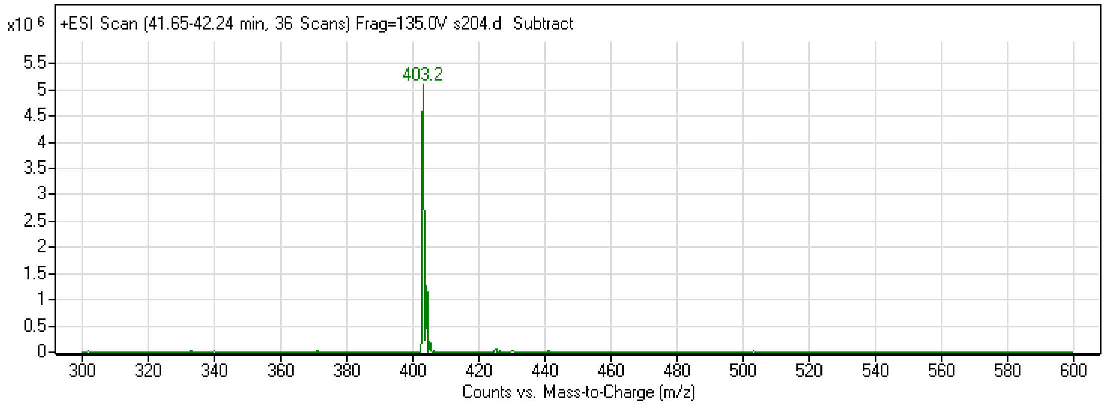

S04

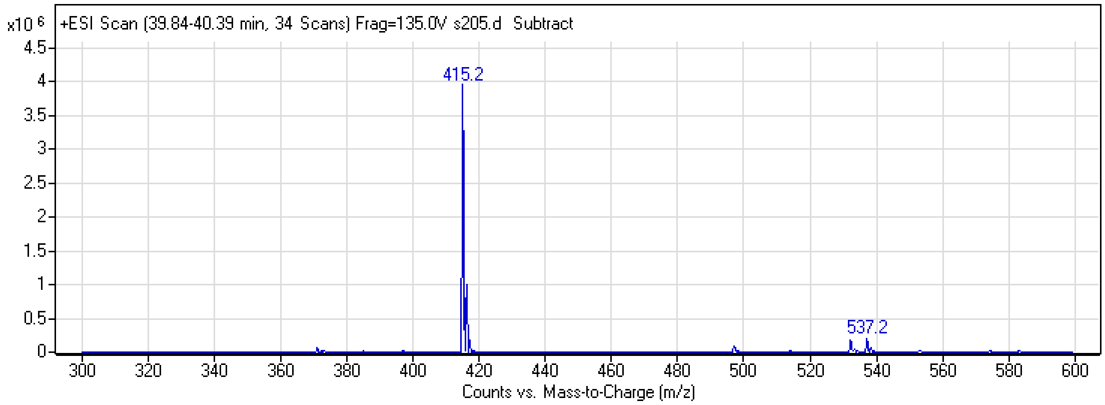

S05

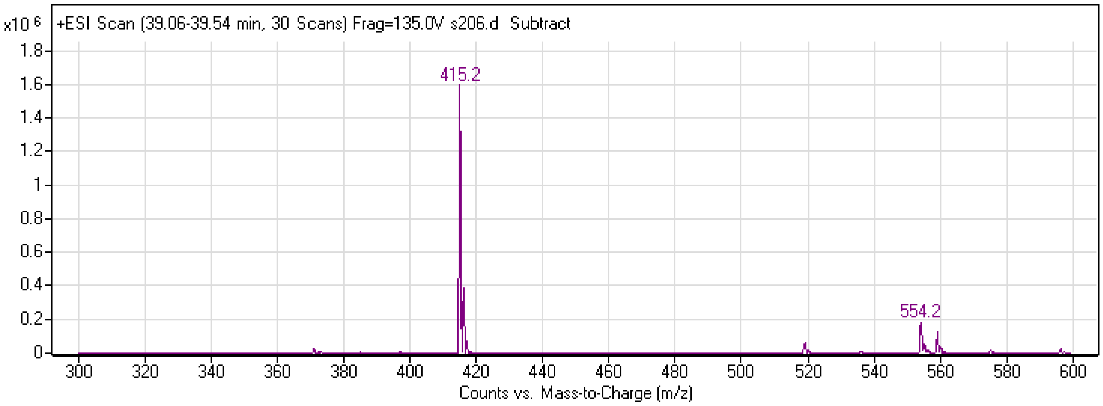
x10 6
+ESI Scan (39.06-39.54 min, 30 Scans) Frag=135.0V s206.d Subtract
415.2
554.2
Counts vs. Mass-to-Charge (m/z)

S06

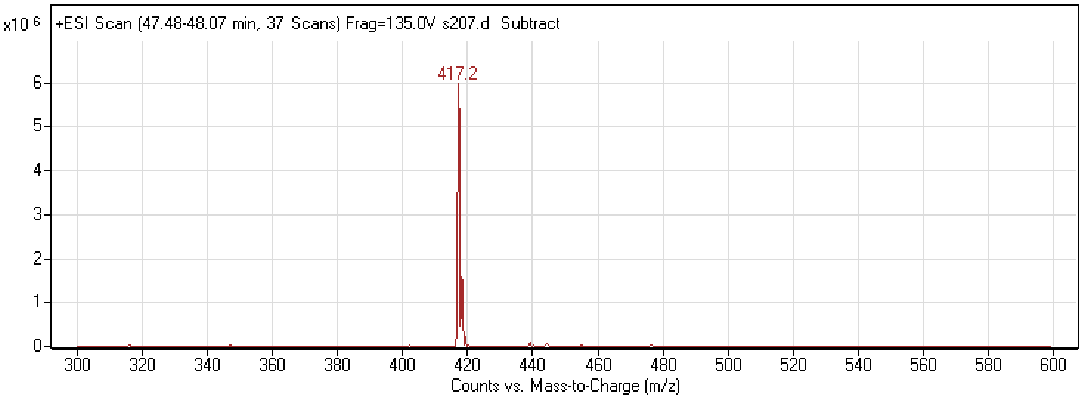
x10 6
+ESI Scan (47.48-48.07 min, 37 Scans) Frag=135.0V s207.d Subtract
417.2
Counts vs. Mass-to-Charge (m/z)

S07

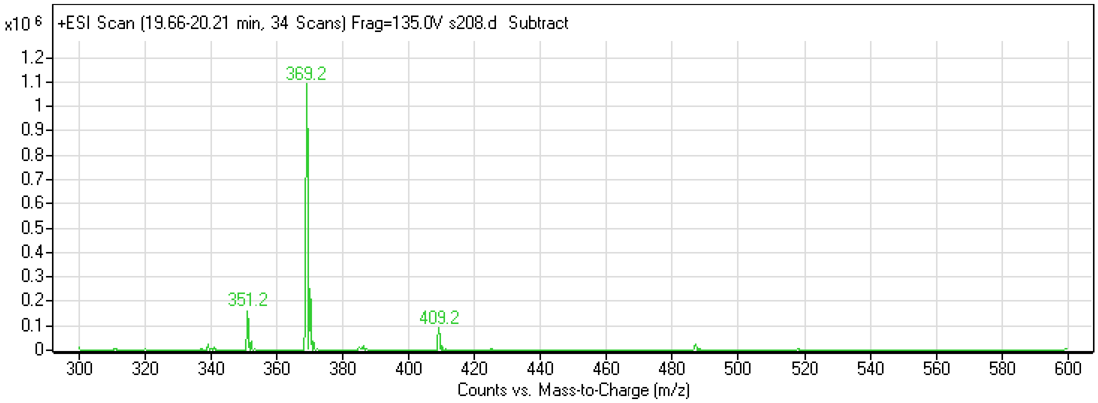
x10 6
+ESI Scan (19.66-20.21 min, 34 Scans) Frag=135.0V s208.d Subtract
369.2
351.2
409.2
Counts vs. Mass-to-Charge (m/z)

S08

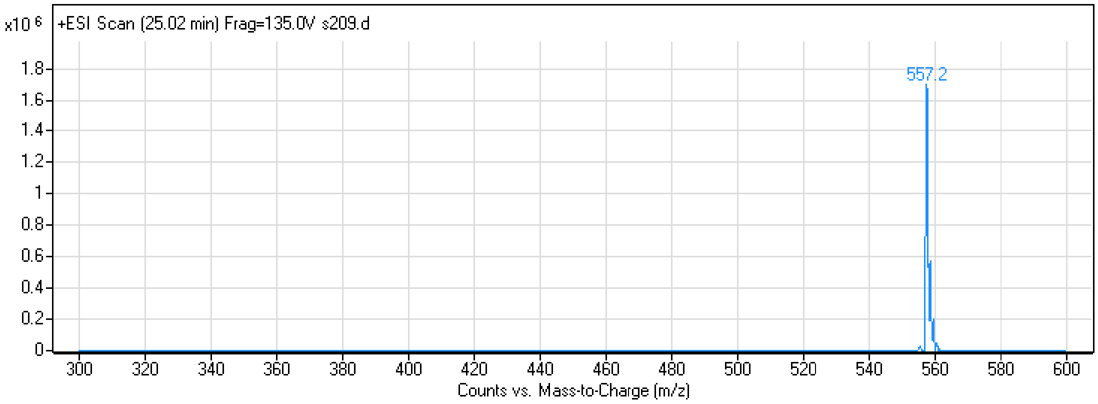
x10 6
+ESI Scan (25.02 min) Frag=135.0V s209.d
557.2
Counts vs. Mass-to-Charge (m/z)

S09

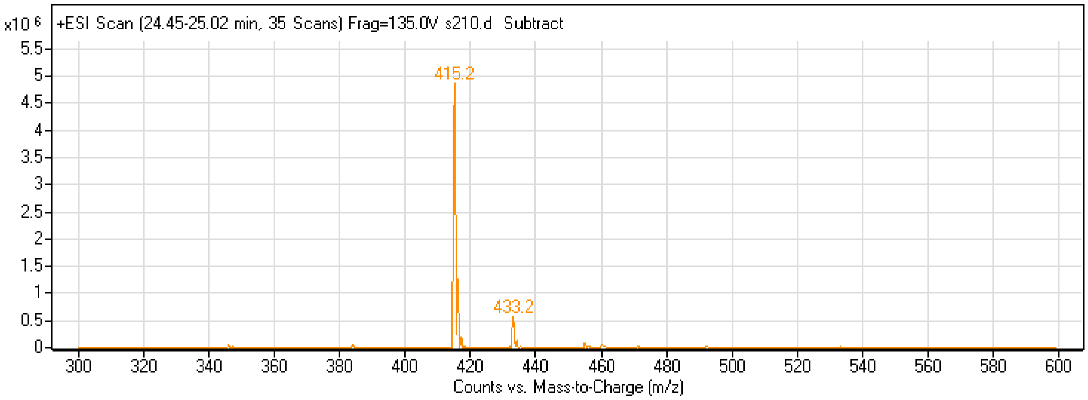

S10

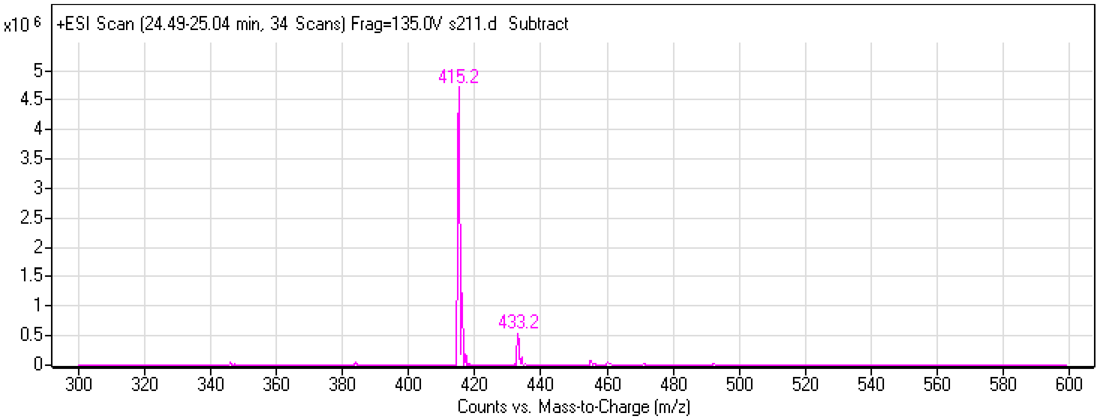

S11

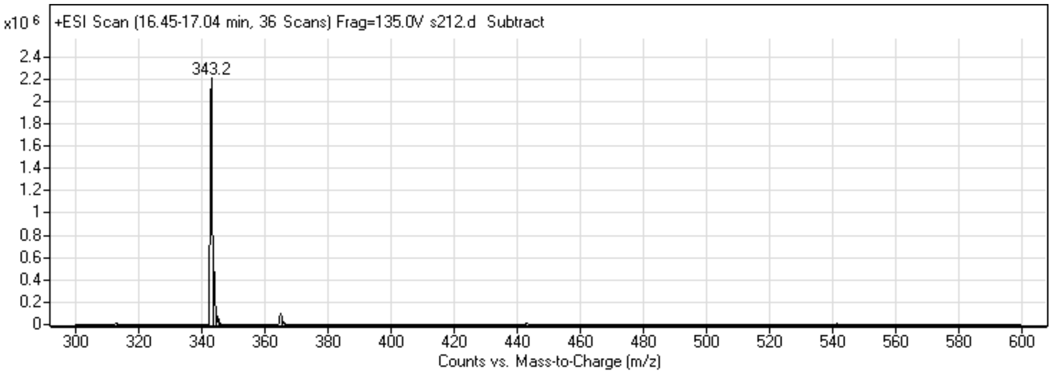

S12

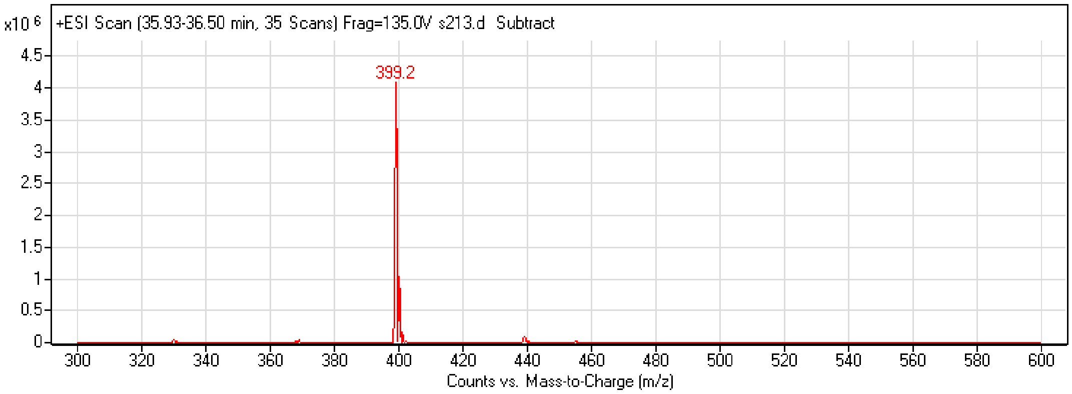

S13

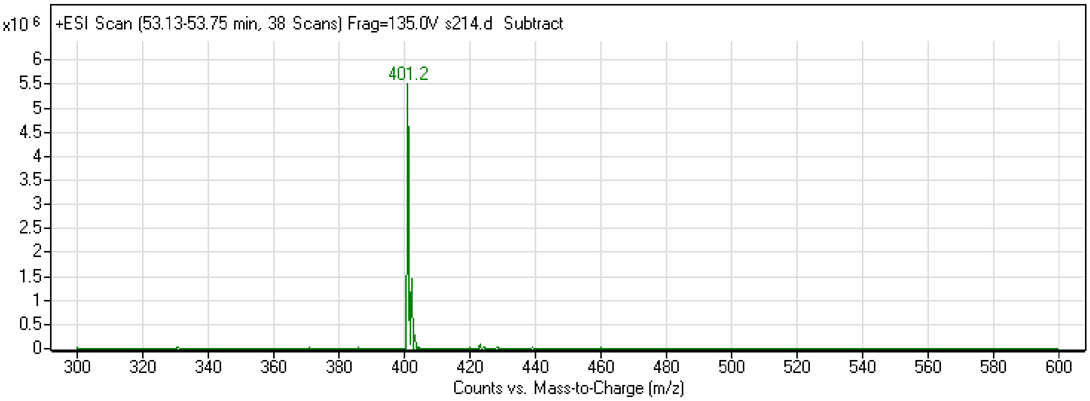

S14

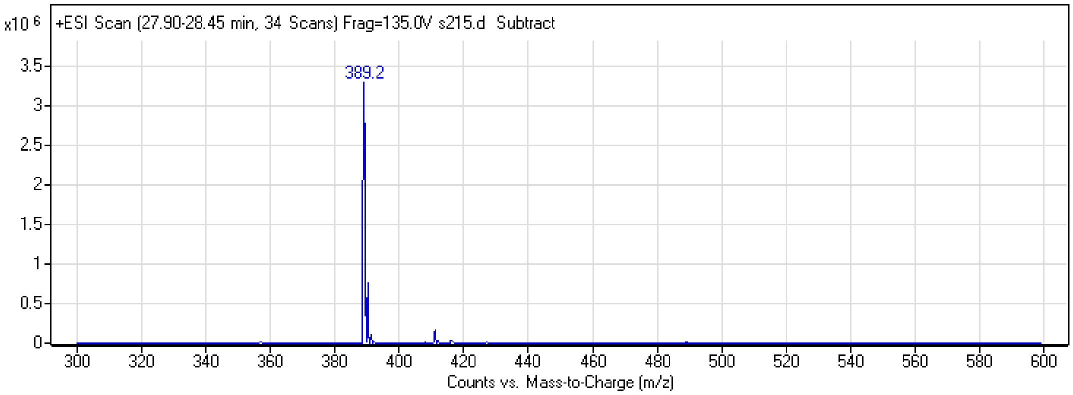

S15

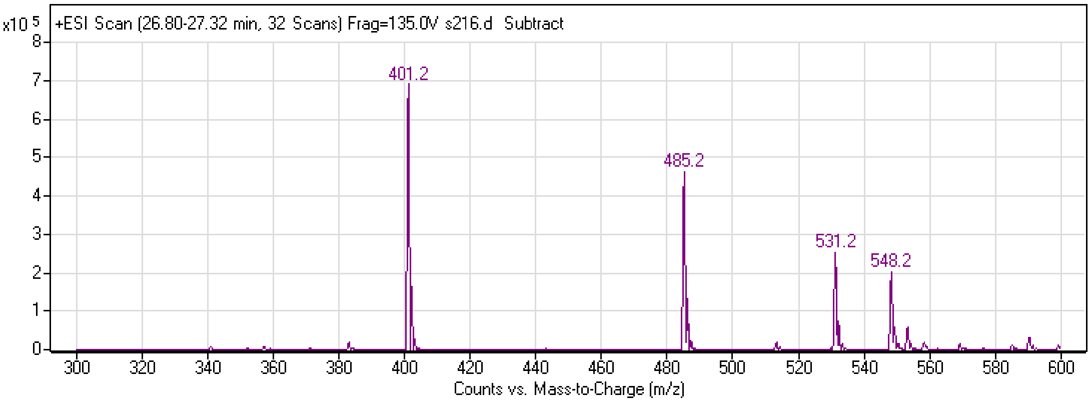

S16

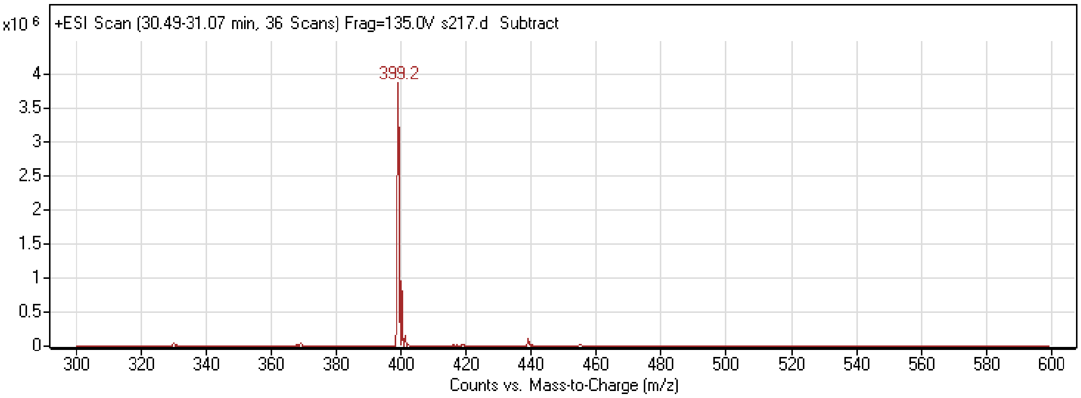

S17

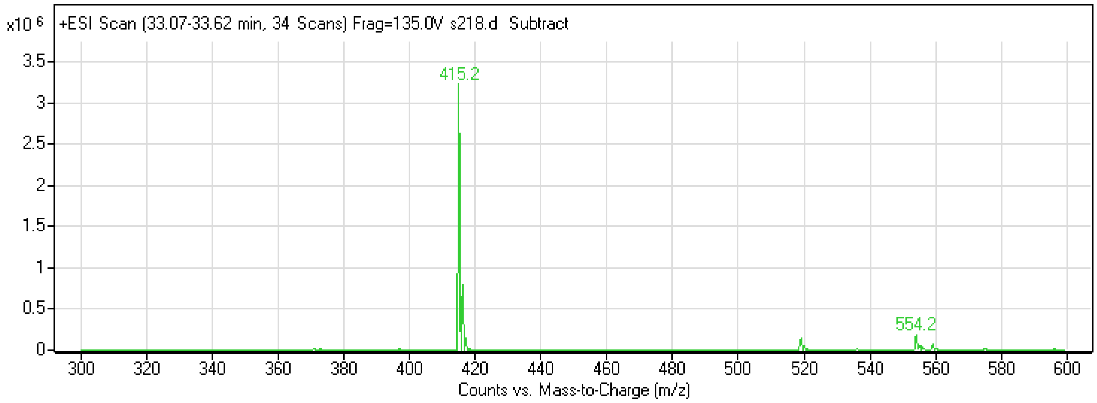

S18

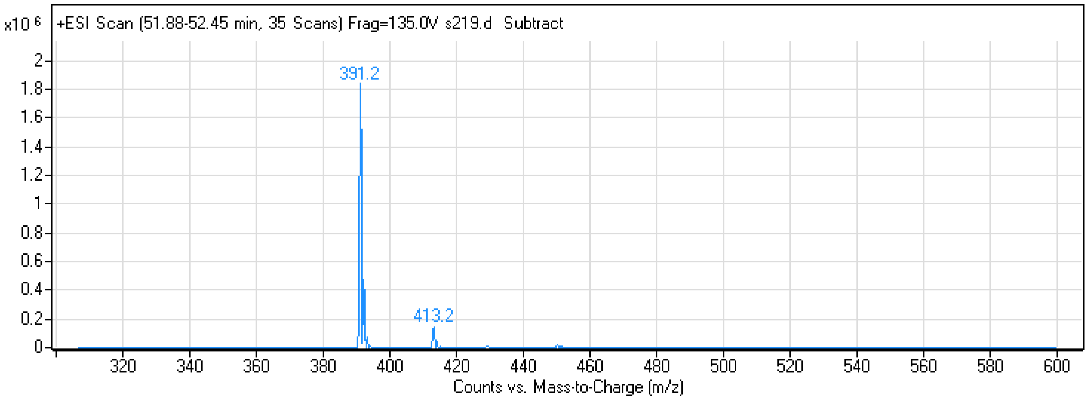

S19

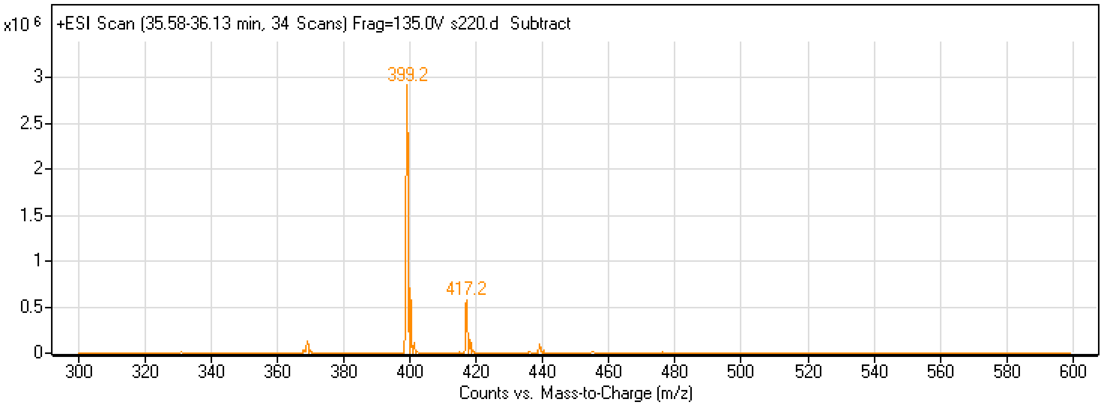

S20

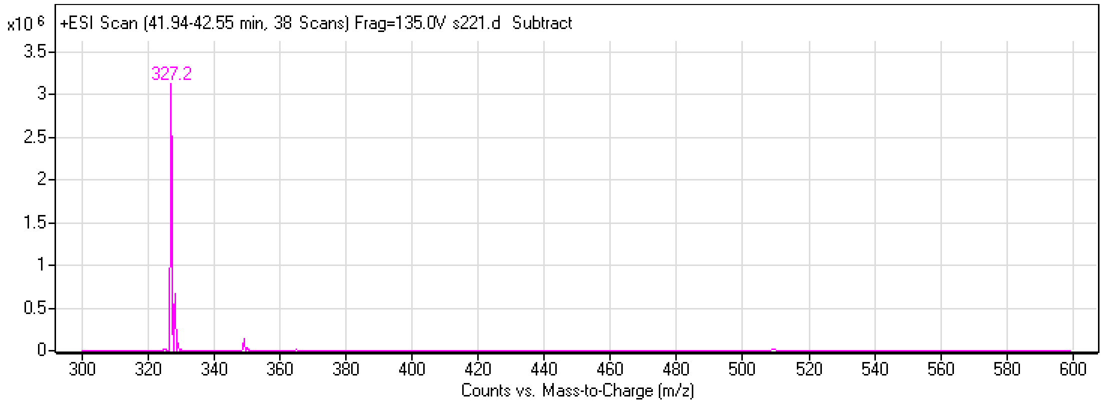
x10 6
+ESI Scan (41.94-42.55 min, 38 Scans) Frag=135.0V s221.d Subtract
327.2
Counts vs. Mass-to-Charge (m/z)

S21

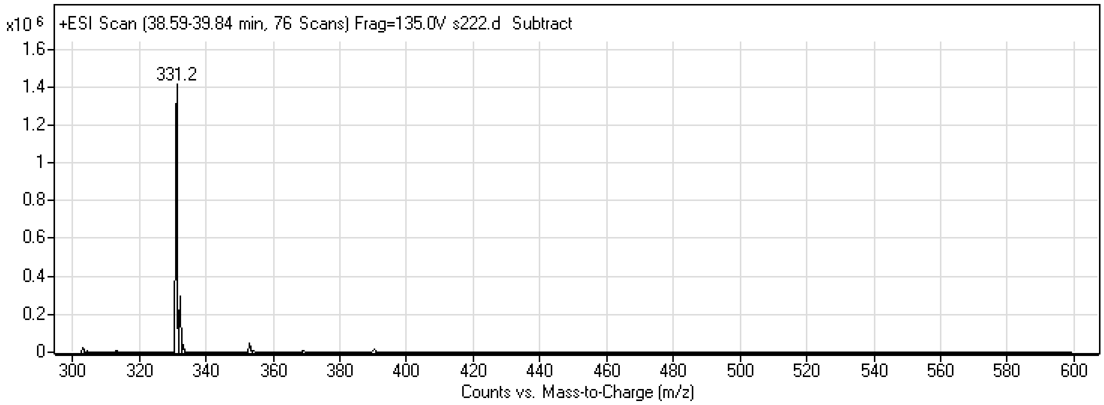
x10 6
+ESI Scan (38.59-39.84 min, 76 Scans) Frag=135.0V s222.d Subtract
331.2
Counts vs. Mass-to-Charge (m/z)

S22

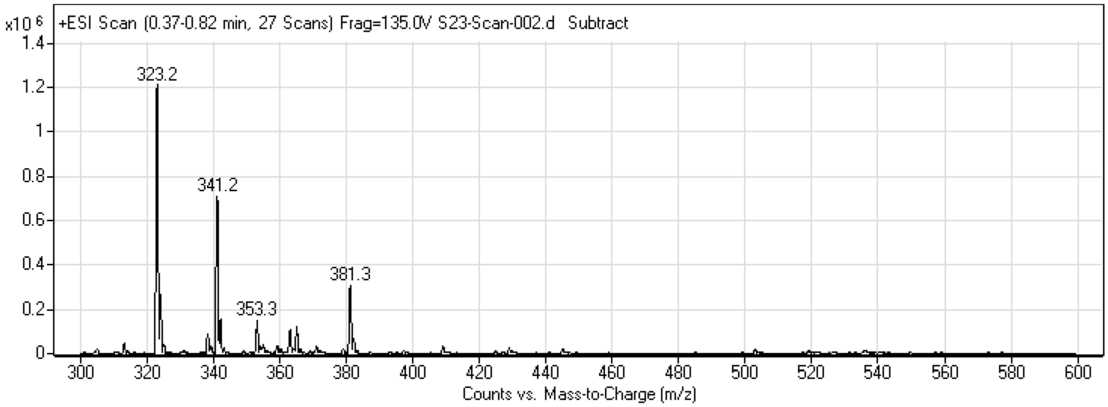
x10 6
+ESI Scan (0.37-0.82 min, 27 Scans) Frag=135.0V S23-Scan-002.d Subtract
323.2
341.2
353.3
381.3
Counts vs. Mass-to-Charge (m/z)

S23

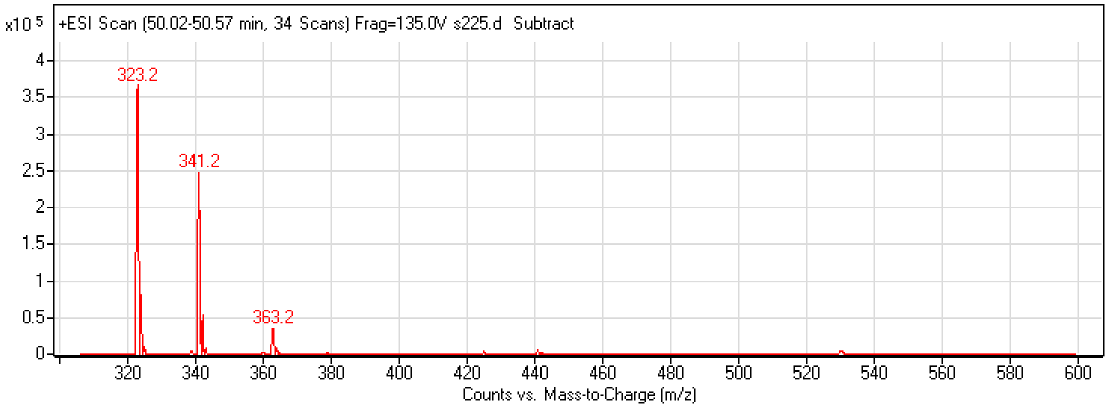
x10 5
+ESI Scan (50.02-50.57 min, 34 Scans) Frag=135.0V s225.d Subtract
323.2
341.2
363.2
Counts vs. Mass-to-Charge (m/z)

S25

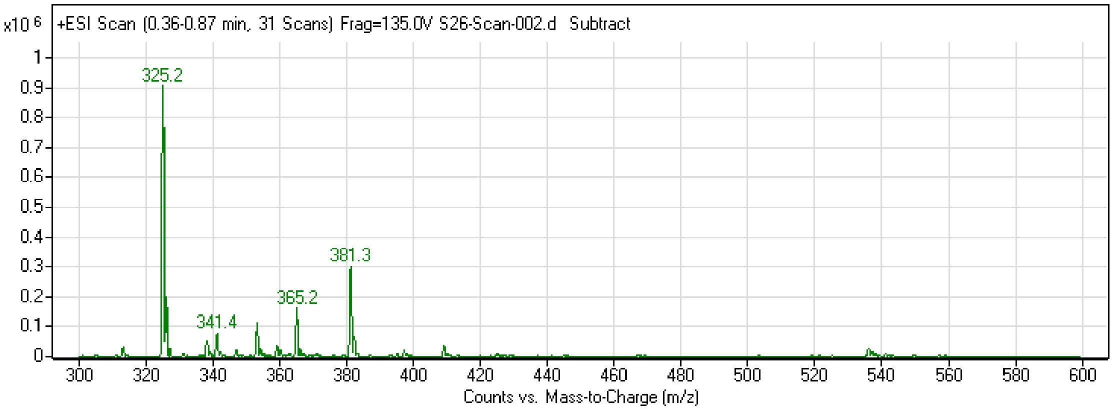

S26

Figure S1. Mass spectra of lignan standards in full scan mode. Ionization conditions applied: positive ionization (+ESI), drying gas temperature - 350°C, gas flow - 12 l/min, nebulizer pressure - 35 psi.

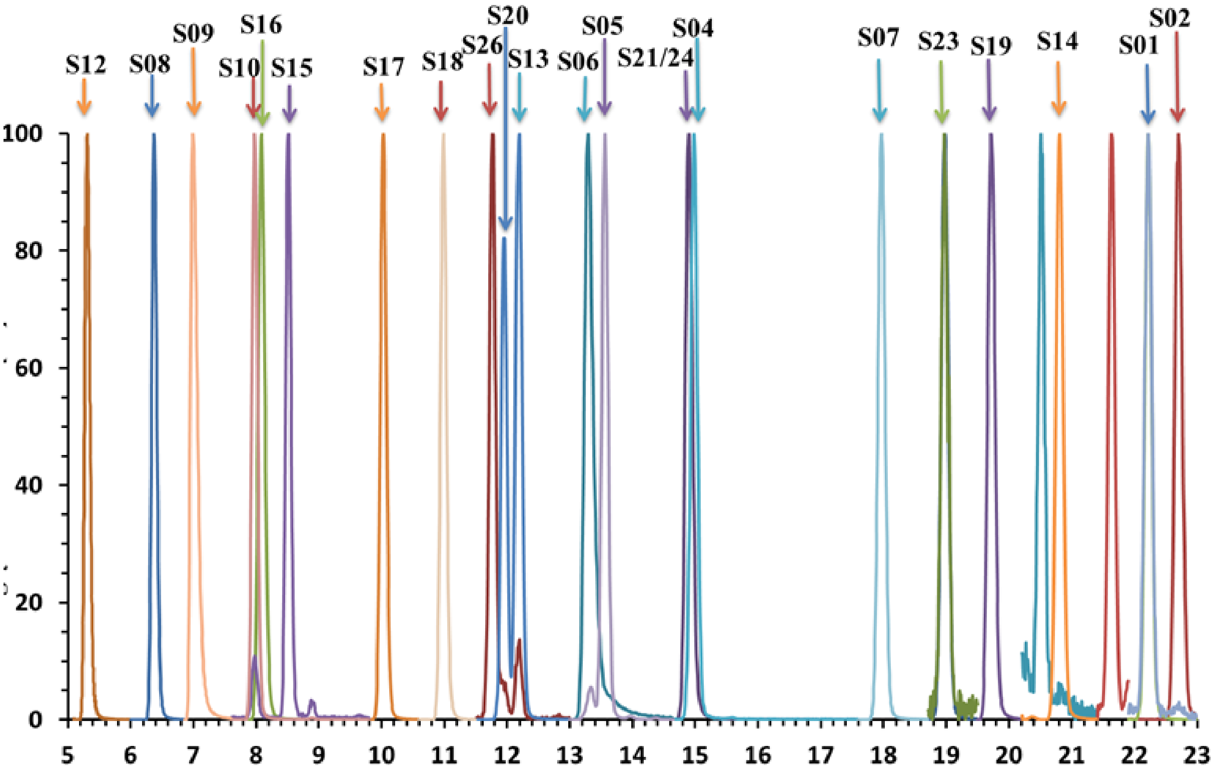

Figure S2. Chromatogram of standard mixture.

Table S1. Monitored lignans and selected experimental parameters

Lignan	Short name	Type of ion	RT [min] C18 column	RT window [min]	MRM transition	RSD% for peaks areas
Wulignan A_1	**S12**	[M+H]+	5.31	0.1	343.3\117.0	20
Rubrisandrin A	**S08**	[M-19]+	6.37	0.1	369.3\351.1	20
Rubriflorin A	**S10**	[M+H]+	6.99	0.1	557.4\173.0	20
Schisandrin	**S09**	[M-H2O+H]+	7.97	0.1	415.3\359.2	20
Gomisin D	**S16**	[M-C6H10O3+H]+	8.52	0.1	401.3\168.0	20
Gomisin J	**S15**	[M+H]+	8.90	0.1	389.3\117.0	20
Pregomisin	**S17**	[M+H]+	19.71	0.1	391.3\139.0	20
Gomisin N	**S18**	[M+H]+	20.81	0.1	401.3\168.0	20
Gomisin A	**S20**	[M-H2O+H]+	10.03	0.1	399.3\368.2	20
Epigomisin O	**S13**	[M-H2O+H]+	11.95	0.1	399.3\368.2	20
6-O-Benzoylgomisin O	**S22**	[M-C7H6O2+H]+	22.22	0.1	399.3\368.2	20
Gomisin G	**S06**	[M-C7H6O2+H]+	10.99	0.1	415.3\371.1	20
Schisantherin A	**S05**	[M-C7H6O2+H]+	13.56	0.1	415.3\371.1	20
Schisantherin B	**S04**	[M-C5H8O2+H]+	13.97	0.1	415.3\371.1	20
Licarin B	**S21**	[M+H]+	11.75	0.1	325.2\152.0	20
Gomisin O	**S07**	[M-H2O+H]+	12.20	0.1	399.3\368.2	20
Mesodihydroguaiaretic acid	**S25**	[M+H]+	13.29	0.1	331.3\117.0	20
Dehydroisoeugenol	**S19**	[M+H]+	14.90	0.1	327.3\105.1	20
Schisanhenol	**S14**	[M+H]+	14.98	0.1	403.3\231.0	20
Schisandrin A	**S01**	[M+H]+	17.97	0.1	417.3\316.2	20
Fragransin A_2	**S02**	[M-21]+	18.98	0.1	323.2\152.0	20
Schisandrin C	**S23**	[M+H]+	22.70	0.1	385.3\231.1	20

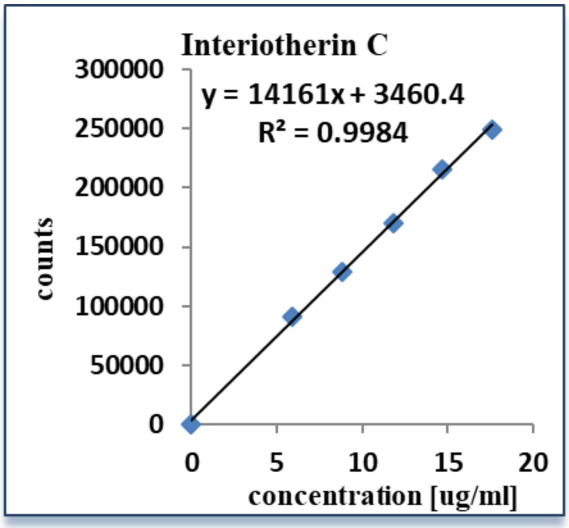
Interiotherin C
y = 14161x + 3460.4
R² = 0.9984
counts
concentration [ug/ml]

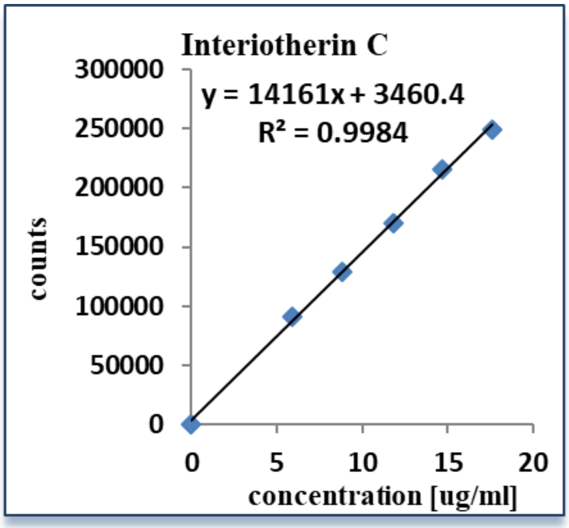
Interiotherin C
y = 14161x + 3460.4
R² = 0.9984
counts
concentration [ug/ml]

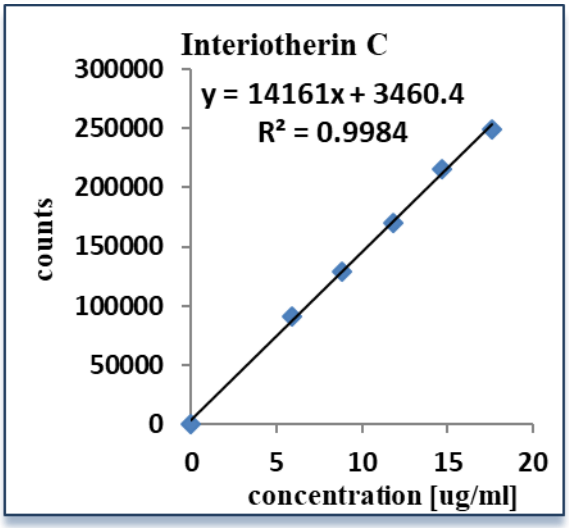
Interiotherin C
y = 14161x + 3460.4
R² = 0.9984
counts
concentration [ug/ml]

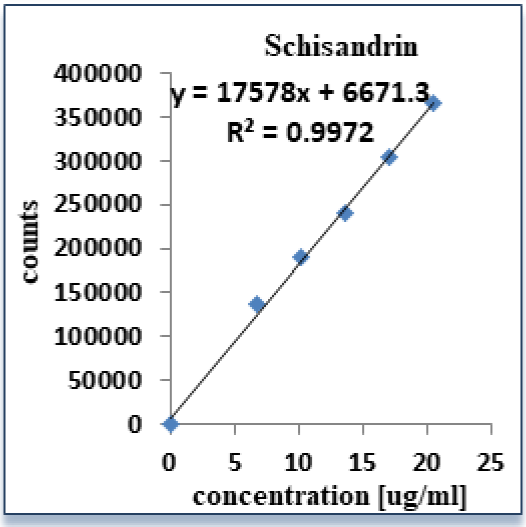
Schisandrin
y = 17578x + 6671.3
R² = 0.9972
counts
concentration [ug/ml]

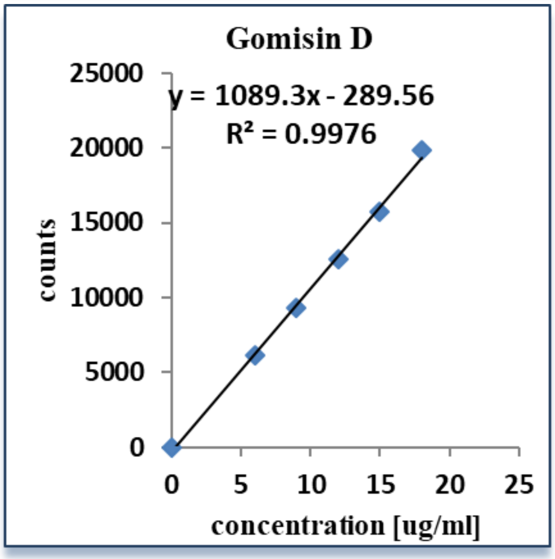
Gomisin D
y = 1089.3x - 289.56
R² = 0.9976
counts
concentration [ug/ml]

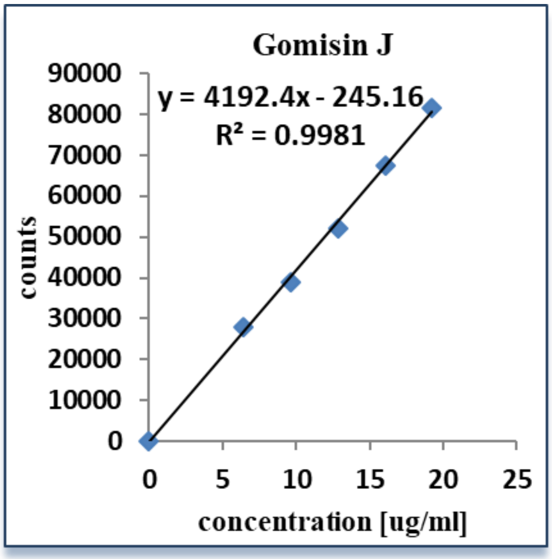
Gomisin J
y = 4192.4x - 245.16
R² = 0.9981
counts
concentration [ug/ml]

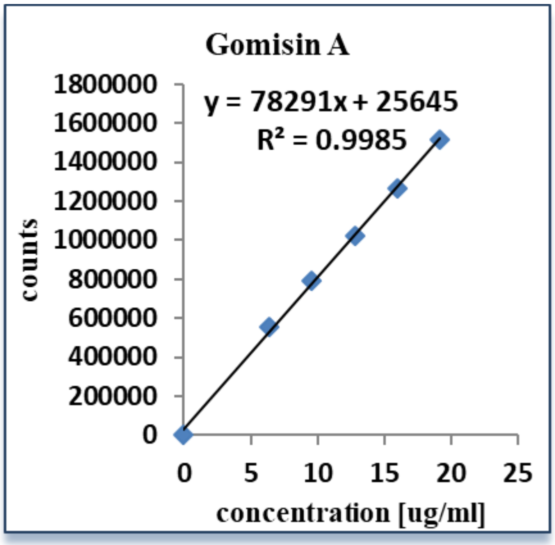
Gomisin A
y = 78291x + 25645
R² = 0.9985
counts
concentration [ug/ml]
0
200000
400000
600000
800000
1000000
1200000
1400000
1600000
1800000
0 5 10 15 20 25

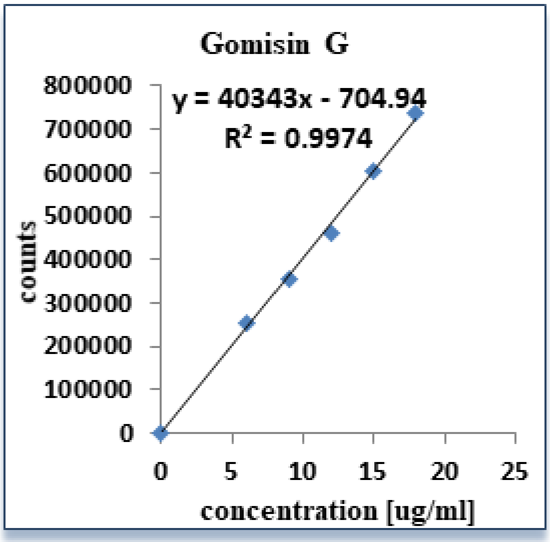
Gomisin G
y = 40343x - 704.94
R² = 0.9974
counts
concentration [ug/ml]
0
100000
200000
300000
400000
500000
600000
700000
800000
0 5 10 15 20 25

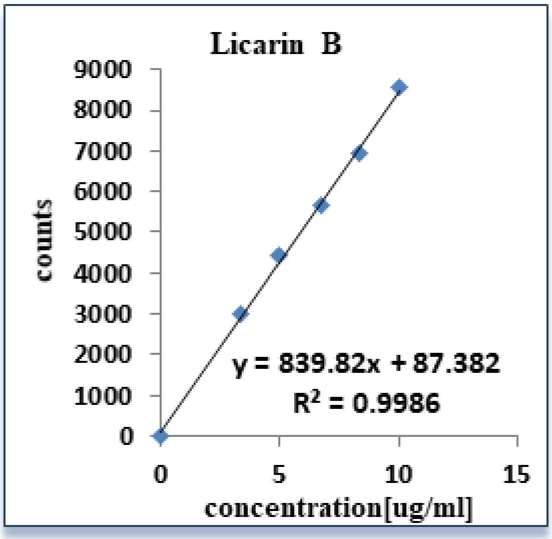
Licarin B
y = 839.82x + 87.382
R² = 0.9986
counts
concentration[ug/ml]
0
1000
2000
3000
4000
5000
6000
7000
8000
9000
0 5 10 15

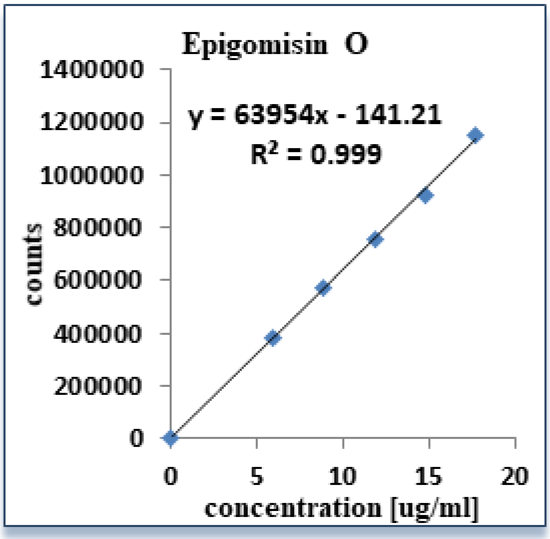
Epigomisin O
y = 63954x - 141.21
R² = 0.999
counts
concentration [ug/ml]
0
200000
400000
600000
800000
1000000
1200000
1400000
0 5 10 15 20

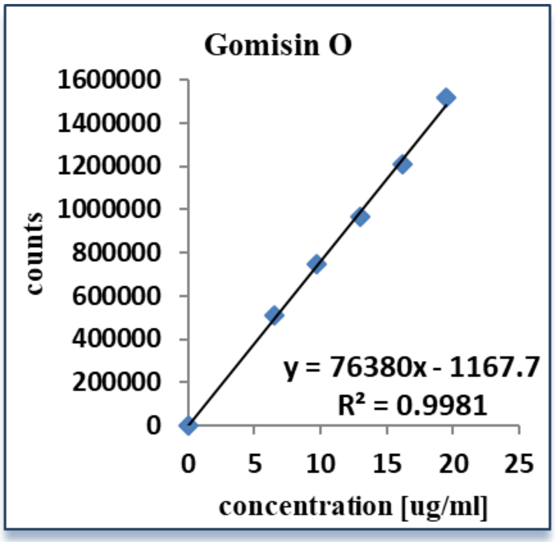
Gomisin O
y = 76380x - 1167.7
R² = 0.9981
counts
concentration [ug/ml]
0
200000
400000
600000
800000
1000000
1200000
1400000
1600000
0 5 10 15 20 25

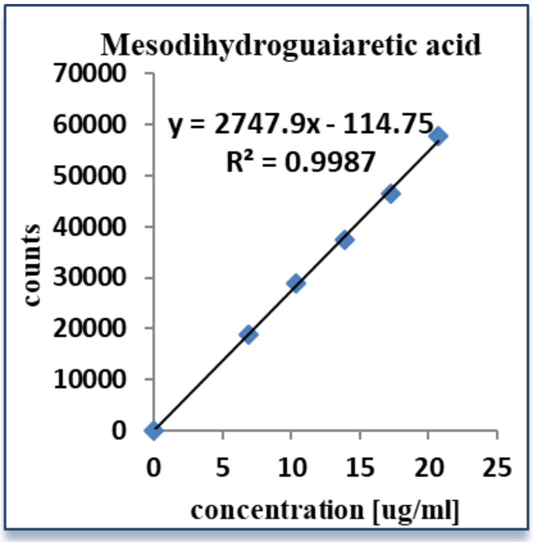
Mesodihydroguaiaretic acid
y = 2747.9x - 114.75
R² = 0.9987
counts
concentration [ug/ml]
0
10000
20000
30000
40000
50000
60000
70000
0 5 10 15 20 25

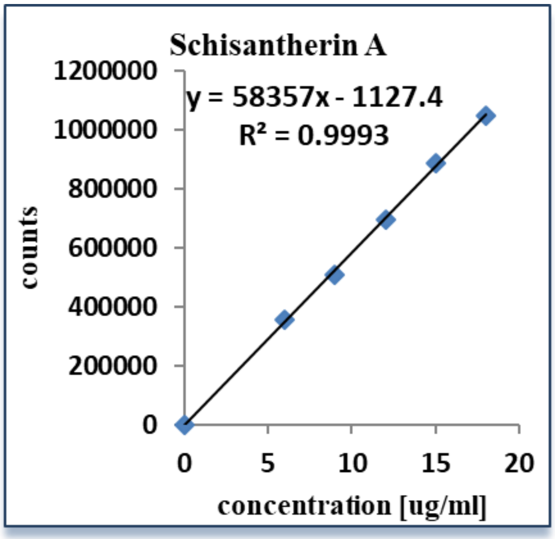
Schisantherin A
y = 58357x - 1127.4
R² = 0.9993
counts
concentration [ug/ml]

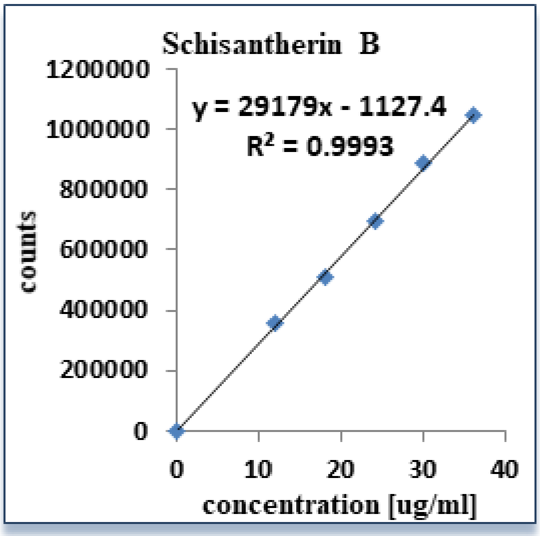
Schisantherin B
y = 29179x - 1127.4
R² = 0.9993
counts
concentration [ug/ml]

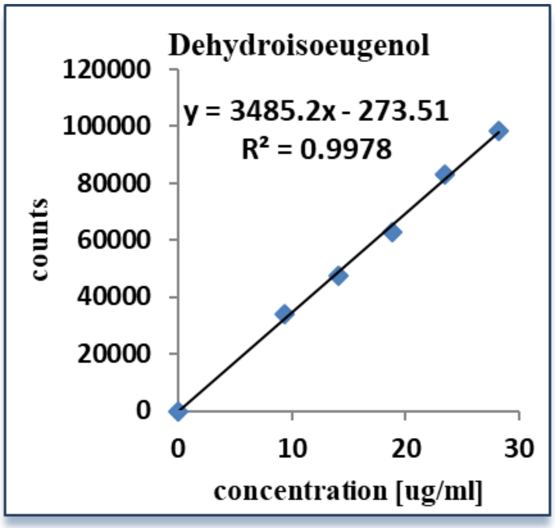
Dehydroisoeugenol
y = 3485.2x - 273.51
R² = 0.9978
counts
concentration [ug/ml]

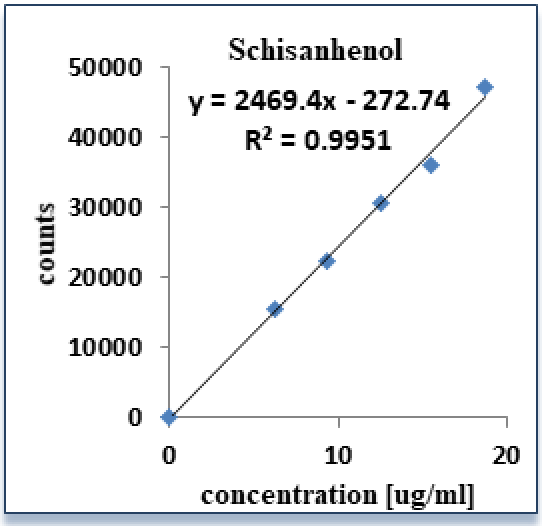
Schisanhenol
y = 2469.4x - 272.74
R² = 0.9951
counts
concentration [ug/ml]

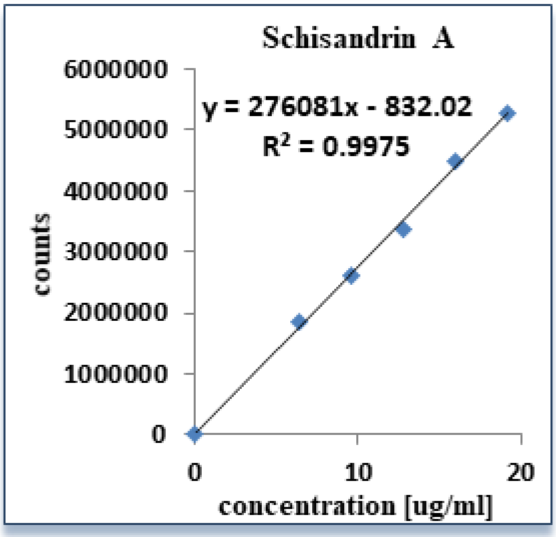
Schisandrin A
y = 276081x - 832.02
R² = 0.9975
counts
concentration [ug/ml]

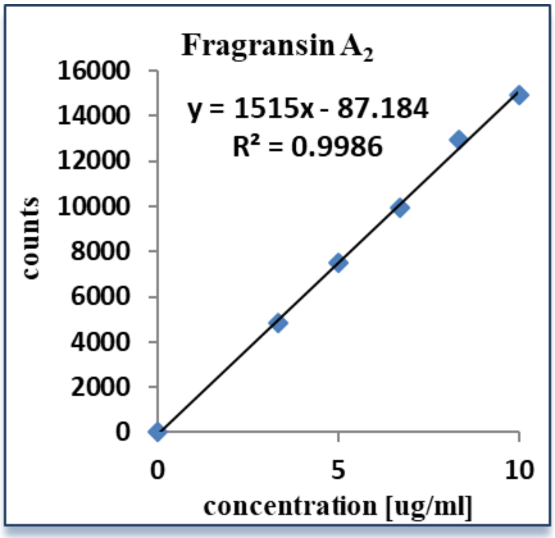
Fragransin A2
y = 1515x - 87.184
R² = 0.9986
counts
concentration [ug/ml]

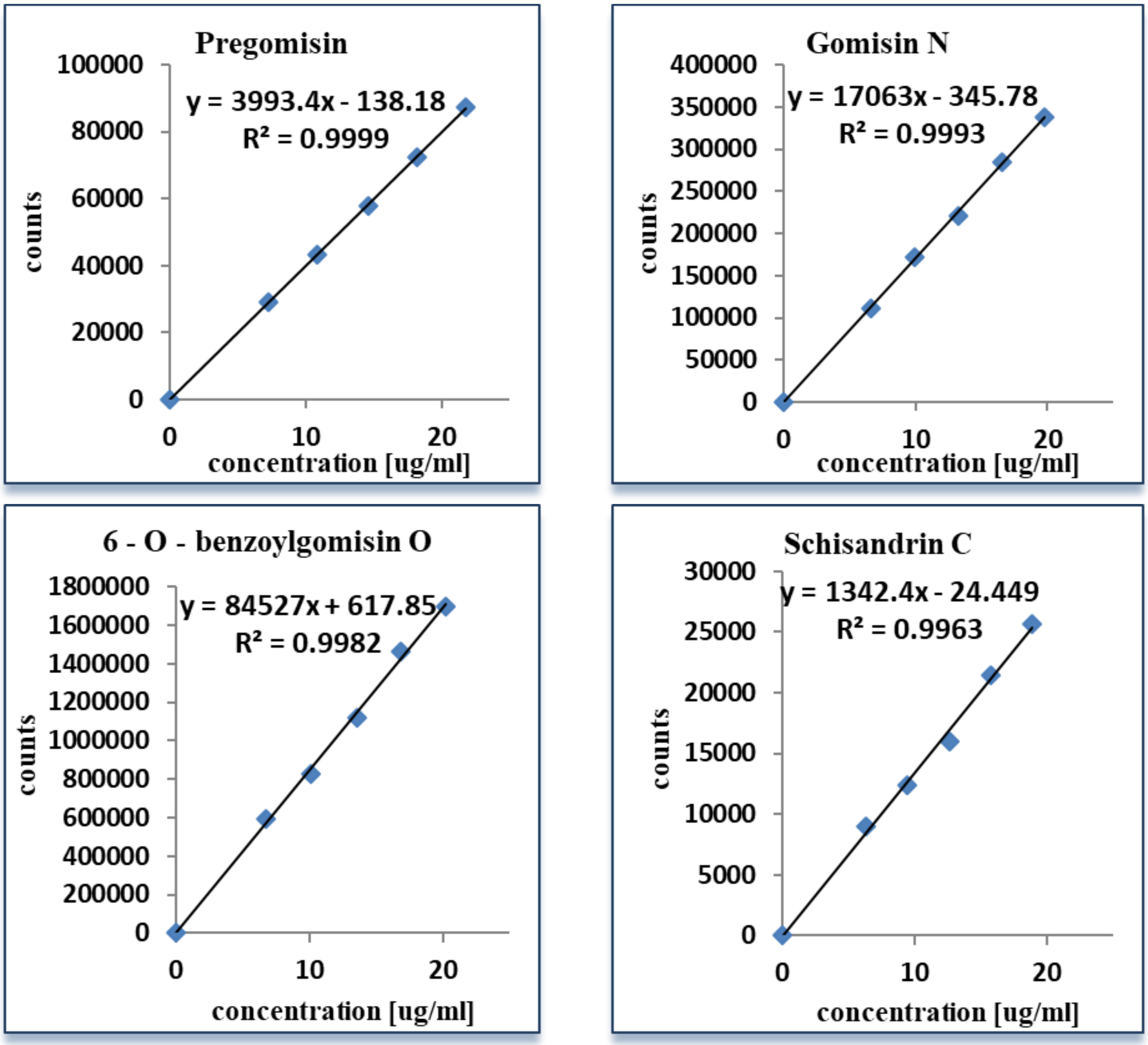

Figure S3. Calibration linearity functions for monitored compounds
